# Chemical and Mechanical Characterization of Unprecedented Transparent Epoxy–Nanomica Composites—New Model Insights for Mechanical Properties

**DOI:** 10.3390/polym15061456

**Published:** 2023-03-15

**Authors:** Greta Ongaro, Alessandro Pontefisso, Elena Zeni, Francesco Lanero, Alessia Famengo, Federico Zorzi, Mirco Zaccariotto, Ugo Galvanetto, Pietro Fiorentin, Renato Gobbo, Roberta Bertani, Paolo Sgarbossa

**Affiliations:** 1 Department of Structural and Geotechnical Engineering, Sapienza University of Rome, v. A. Gramsci 53, 00197 Rome, Italy; 2 Department of Management and Engineering, University of Padova, Stradella San Nicola 3, 36100 Vicenza, Italy; 3 Department of Industrial Engineering, University of Padova, v. Gradenigo, 6, 35131 Padova, Italy; 4 National Research Council of Italy (CNR), Institute of Condensed Matter Chemistry and Technologies for Energy, Corso Stati Uniti 4, 35127 Padova, Italy; 5 Centro di Analisi e Servizi per la Certificazione (CEASC), University of Padova, Via Jappelli 1/A, 35131 Padova, Italy

**Keywords:** micas, epoxy nanocomposites, environmental scanning electron microscopy, mechanical, thermal, optical and dielectric properties, peridynamics-based model

## Abstract

Two nanomicas of similar composition, containing muscovite and quartz, but with different particle size distributions, have been used to prepare transparent epoxy nanocomposites. Their homogeneous dispersion, due to the nano-size, was achieved even without being organically modified, and no aggregation of the nanoparticles was observed, thus maximizing the specific interface between matrix and nanofiller. No exfoliation or intercalation has been observed by XRD, despite the significant dispersion of the filler in the matrix which produced nanocomposites with a loss in transparency in the visible domain of less than 10% in the presence of 1% wt and 3% wt of mica fillers. The presence of micas does not affect the thermal behavior of the nanocomposites, which remains similar to that of the neat epoxy resin. The mechanical characterization of the epoxy resin composites revealed an increased Young’s modulus, whereas tensile strength was reduced. A peridynamics-based representative volume element approach has been implemented to estimate the effective Young’s modulus of the nanomodified materials. The results obtained through this homogenization procedure have been used as input for the analysis of the nanocomposite fracture toughness, which has been carried out by a classical continuum mechanics–peridynamics coupling approach. Comparison with the experimental data confirms the capability of the peridynamics-based strategies to properly model the effective Young’s modulus and fracture toughness of epoxy-resin nanocomposites. Finally, the new mica-based composites exhibit high values of volume resistivity, thus being excellent candidates as insulating materials.

## 1. Introduction

The development of new polymeric materials combining different and/or specific properties, such as mechanical strength, biodegradability, and recyclability is still a priority objective in many industrial areas, due to its challenges. Polymer-layered silicate nanocomposites have attracted great attention in both the academic and industrial fields, since many important properties can be improved by design, introducing a small amount of nanosized clay particles (environmentally friendly fillers) into the polymer matrix. One of the most extensively applied strategies to achieve it is the modification of the clay with organic molecules so that the clay surface becomes organophilic [1,2,3,4,5].

Epoxy resins find many industrial applications [6,7] and are particularly suitable for preparing nanocomposites with layered silicates due to the polarity of the epoxy monomers which can favor the compatibilization with the silicates and diffuse easily into the clay cavities [8,9,10,11]. In fact, the level of dispersion of the filler in the matrix is one of the crucial parameters to determine any enhancement of the properties of the nanocomposites [12,13,14] and is related to the nature of the curing agent, as well as the curing conditions and the dispersion procedure.

Between layered silicates, the addition of natural or synthetic mica to a polymeric matrix was reported to improve mechanical, dielectric, and thermal properties, as well as sound absorption [15]. The introduction of mica nanoparticles (5% wt) led to an increase in the elastic modulus of the polystyrene-based composite, with a reduction in the elongation at break and the maintenance of strength at the level of pure polystyrene [16]. Environmentally compatible, halogen-free, mica-based coatings were successfully deposited on flexible polyurethane foam (PUF) giving rise to layer-by-layer assemblies, which, when exposed to a crude flame from a butane torch, preserved the inner foam by forming a char able to protect underlying foam [17]. It is noteworthy that a high-performance (low density, high strength, and high fracture toughness) nacreous aramidic mica bulk material can be obtained via a mild controlled and scalable bottom-up assembly strategy [18].

Even if micas do not expand in water due to the strong electrostatic interaction between the layer’s negative charge and the interlayer K^+^ ions [19], to improve the dispersion and compatibilization with the polymeric matrix, organic (i.e., with ethyl-vinyl-acetate [20] or diacetone-acrylamide [21]), organometallic (i.e., with triethylaluminum [22]) or inorganic (i.e., exchanging with metal cations [23,24]) modifications together with the addition of different coupling agents (i.e., maleic anhydride [25] or silane [26]) have been investigated.

The high aspect ratio (lateral length/thickness ratio > 50) and platelet shape characterize the micas as fillers and influence the physical properties of nanocomposites in a different way with respect to spherical particles (SiO_2_ and Al_2_O_3_) [27,28].

Numerous examples of mica–epoxy resin nanocomposites have been reported in the literature, yielding materials for different applications [29,30,31] with improved thermal conductivity, dielectric loss, and mechanical properties. They are commonly prepared by direct mixing and solution mixing, improved by shear devices such as extruders, mixers, or ultrasonicators [2,32].

To study their specific technological properties, the common mechanical, thermal, and electrical tests can be applied, with the aim of understanding the relationship between properties and structure/composition. In this respect, numerical analysis is an essential tool for characterizing the properties of various materials and modeling their performance. Recently, several numerical approaches based on classical continuum mechanics (CCM) have been employed to these ends [33,34,35]. Innovative computational approaches based on the peridynamic (PD) theory, which is a nonlocal reformulation of classical continuum mechanics based on integrodifferential equations, have recently been proposed to address the shortcomings of CCM-based methods [36]. Considering that the theory deals with integral equations rather than spatial differentiation, PD-based strategies can handle material discontinuities, thus allowing for the modeling of the interfaces between different phases and for the treatment of fracture and failure as a natural material response [37]. Moreover, the introduction of a length parameter allows for modeling material behavior at different length scales, thus making the theory suited also to the study of nanocomposites [38]. However, PD-based models are computationally more expensive than CCM-based ones due to their nonlocal nature, which hinders their application in large-scale simulations. Moreover, since the application of boundary conditions in PD is nonlocal, it is more challenging than that in the classical theory framework. Hence, it is convenient to couple computational methods based on classical continuum mechanics with those based on peridynamics [39,40,41,42].

In this work, two nanomicas of similar composition but with different particle size distributions, Mica 10 and Mica 45, have been used to prepare epoxy nanocomposites with different filler content. Their homogeneous dispersion, due to the nano-size, was achieved even without organic modification and without the aggregation of the nanoparticles, thus maximizing the specific interface between the matrix and the nanofiller and yielding unexpectedly transparent epoxy resin composites. All the new composites have been characterized in terms of mechanical properties (tensile strength and fracture toughness), thermal stability, and optical and electrical properties. Subsequently, the multiscale PD framework recently proposed in [38] was exploited to model the mechanical properties of the new materials. A PD-based representative volume element (RVE) approach was here implemented to estimate the effective Young’s modulus of the nanomodified epoxy resins. The results obtained through this homogenization procedure were then used as input for the analysis of the nanocomposite fracture toughness, which was carried out by exploiting a classical continuum mechanics–peridynamics (CCM-PD) coupling approach. The PD-based approaches were calibrated by exploiting the data from the tensile and fracture tests carried out in this work.

The paper is organized as follows. In Section 2, the preparation of the mica–epoxy nanocomposites and the methods exploited for the characterization of their morphological, mechanical, thermal, optical, and resistive properties are comprehensively described. Section 3 discusses the results obtained through the experimental material characterization. In Section 4, the RVE-based approach and the coupling-based strategy implemented to model the effective Young’s modulus and fracture toughness of the materials are presented. Section 5 closes the paper with some remarks and proposals for future research.

## 2. Materials and Methods

Nanomicas (Mica 10 and Mica 45) have been kindly supplied by Veneta Mineraria S.p.a. (Este, Italy) and used as received. A diglycidyl ether of bisphenol A epoxide (DGEBA, Elan-tech EC157) and a mixture of cycloaliphatic amines (Elan-tech W152LR), both supplied by Elantas Europe (Collecchio, Italy), were employed as polymer precursors.

### 2.1. Preparation of Epoxy/Nanomicas Composites

**Scheme 1:**
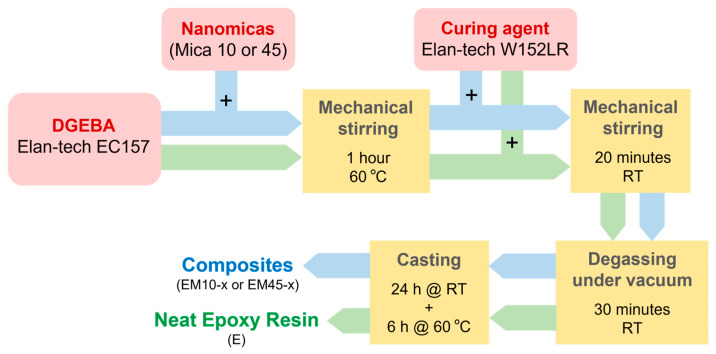
Preparation procedure for all the samples.

The nanocomposites were prepared as depicted in Scheme 1 through mechanical dispersion by mixing the epoxy resin with the nanomicas. Specifically, epoxy formulations containing 1, 3, 5, 10, and 15% by weight of micas were considered, as summarized in Table 1. Initially, the measured amount of mica was added to the diglycidyl ether of bisphenol A epoxide (120 g) in a flask. The dispersion process was carried out at 60 °C under mechanical stirring for about 1 h until the dispersion was homogeneous. Then, after cooling at room temperature, the curing agent was added (40 g) to the suspension under mechanical stirring for 20 min, according to the stoichiometric ratio from the supplier’s datasheet (3/1 = epoxy/amine wt/wt). The reaction mixture was degassed for about 30 min after the addition of the hardener to remove the air trapped within the blend. Finally, the obtained mixture was cast onto open silicon molds to be cured at room temperature for 24 h and then in an oven at 60 °C for 6 h.

### 2.2. Characterization of the Micas and of the Nanocomposites

Fourier transform infrared spectroscopy (FTIR) spectra were obtained on a Perkin Elmer 100 in ATR mode (diamond crystal). For each sample, 32 scans were recorded in the range of 4000–400 cm^−1^.

Powder and nanocomposite XRD analyses were carried out with a PANanalytical X’Pert 3 Powder diffractometer equipped with a Cu X-Ray tube and a real-time multiple strip (RTMS) detector (X’Celerator). The diffraction profiles were collected over the 2θ range of 2.5–85°, with a virtual 2θ step size of 0.013° and a counting time of 100 s/step.

The microanalysis and morphology of the samples have been studied by FEI Quanta 200 ESEM equipped with an EDAX EDX detector. The samples have been observed directly on cross-sections obtained by brittle fracture at the temperature of liquid nitrogen.

Solid-state NMR experiments were collected on a Bruker AVANCE III spectrometer 300 operating with a magnetic field at 7.0 T corresponding to ^29^Si Larmor frequency of 59.623 MHz and equipped for solid-state analysis in 4 mm-diameter zirconia rotors with Kel-F caps. The magic angle was accurately adjusted prior to data acquisition using KBr. Briefly, ^29^Si chemical shifts were externally referenced to solid tetrakis(trimethylsilyl)silane at 20.91 ppm. The semi-quantitative ^29^Si single-pulse experiments were collected at a spinning frequency of 5 MHz, a recycling delay of 60 s, and 500 transients [43].

The morphology of the nanocomposites was studied by transmission electron microscopy (TEM) with a JEM 2000 EX-II (Jeol, Japan) microscope at 120 kV. Briefly, 120 nm-thick films were obtained by cutting the samples with a PT-PC Power Tome Ultramicrotome at room temperature (RMC Boeckeler Instruments, USA).

Tensile tests on dog-bone (DB) specimens were carried out using an MTS809 servo-hydraulic machine (Eden Prairie, Minnesota, United States) equipped with a 10 kN load cell at a crosshead speed of 2 mm/min and with an MTS 632.29F-30 extensometer. The specimen geometry was chosen according to the ISO 527-2 suggestions [44]. Five specimens were tested for their mica content in order to obtain statistically representative results. In all cases, failure took place close to the limit of the gauge length of the specimens. Fracture tests were carried out on compact tension (CT) specimens whose geometry and size comply with ASTM D5045-99 suggestions [45]. According to previously reported procedures [46], after demolding, specimens were pre-cracked by manual tapping, obtaining artificial short cracks. Then, 10 mm-long cracks were obtained by loading the tapped samples with some zero-to-tension fatigue cycles. For each composite, five specimens were tested. The experimental results were rearranged, and for each test, the value of *K_Ic_* was determined starting from the crack length and the fracture load, according to ASTM D5045-99 [45].

Thermogravimetric analyses (TGA) were carried out on 30–35 mg of samples placed on Al_2_O_3_ crucibles under a N_2_ atmosphere (60 mL/min), heating from RT to 700 °C at 10 °C/min using a Netzsch STA 449 C analyzer (Netzsch-Gerätebau GmbH, Verona, Italy). Data were processed and analyzed by Netzsch Proteus software 6.1.0.

The transparency measurements have been carried out by using the radiance integrating sphere standard OL455 from Optronic Laboratories as a stable light source in the visible range. The diameter of the output port was 38 mm. At that output, the radiance values had a uniformity of 0.5% of the average value. The primary source was a halogen lamp powered assuring short-term stability of the produced radiance equal to 0.5%. The electric current through that lamp was selected so that the output spectral radiance was very close to the emission of a blackbody at 2856 K. A Konica Minolta CS-1000a spectroradiometer (Konica Minolta Sensing Europe, Milano, Italy) was used to measure the spectral radiance, working in the range between 380 nm and 780 nm with a spectral resolution of 1 nm; its bandpass function has a full width at half maximum (FWHM) of about 5 nm, with amplitude uncertainty of 2% and a luminance range in the range from 0.01 cd m^2^ to 80,000 cd m^2^. The measures have high repeatability (1 standard deviation) equal to 0.1% of the reading plus 1 digit. The relative error due to the flatness was measured within 5% in the full operating wavelength range [47]. The uncertainty on the measured spectral transmittance is less than 7%.

The volume resistivity of some nanocomposites was measured according to the procedures described in the standard IEC 62631-3-1:2016 [48]. Due to the expected high values, specimens were prepared with the shape of a plate with parallel measuring areas, having a thin thickness of about 2.5 mm (two different samples have been considered for each nanocomposite). The electrodes were realized with high-conductivity silver paints together with the guard electrode used to reduce the leakage currents. The measurements were carried out at ambient temperature (20 °C) with a Keithley Mod 6487 picoammeter/voltage source at 500 V (Keithley Instruments Inc., Cleveland, Ohio, USA) and after one minute of electrification.

## 3. Results and Discussion

### 3.1. Characterization of the Nanomicas

Micas are layer phyllosilicates whose structure is based either on a brucite-like trioctahedral sheet [Mg_3_O_4_(OH)_2_] or a gibbsite-like dioctahedral sheet [Al_2_O_4_(OH)_2_]. This module is sandwiched between a pair of oppositely oriented tetrahedral sheets forming an M layer, often referred to as the 2:1 (or TOT) layer [49].

Mica 10 and Mica 45 are metamorphic K, Na white micas, whose composition can be described as crystalline solutions among two end-member compositions muscovite [K_2_Al_4_(Al_2_Si_6_O_20_)(OH)_4_] and paragonite [Na_2_Al_4_(AlSi_6_O_20_)(OH)_4_] with some deviations typically shown by rock-forming micas represented by titanium and iron [50].

**Figure 1:**
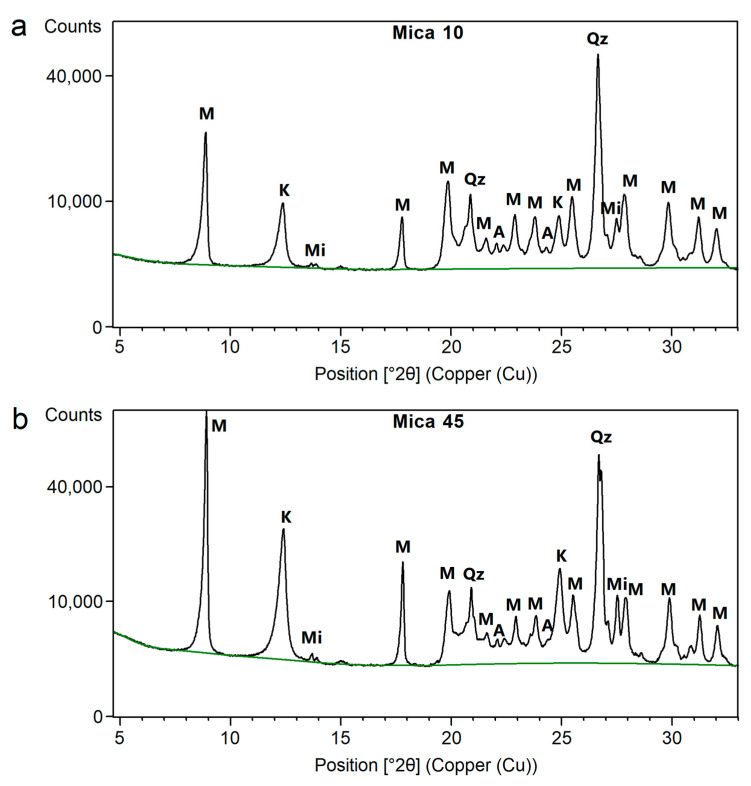
(**a**) XRD of Mica 10; (**b**) XRD of Mica 45 (M = muscovite, K = kaolinite, Mi = microcline, A = albite, Qz = quartz).

In Figure 1, the XRD of Mica 10 and Mica 45 have been reported. The spectra show the mineralogical composition with the presence of muscovite, kaolinite, and quartz as the major components and with microcline and albite to a lower extent. Muscovite is a dioctahedral mica consisting of TOT layers with a highly perfect basal cleavage yielding remarkably thin laminae which are often highly elastic. Kaolinite is a clay mineral, with the chemical composition Al_2_Si_2_O_5_(OH)_4_, consisting of layers formed by sheets of tetrahedral silica linked to sheets of octahedral alumina through oxygen atoms. Albite and microcline are feldspars, a group of aluminum tectosilicate minerals. Albite is the Na-rich term of albite-anorthite series (plagioclase) with the general formula NaAlSi_3_O_8_, whereas microcline is a potassium-rich alkali feldspar with the general formula KAlSi_3_O_8_. Mineralogical quantitative estimates by XRD (Rietveld refinement) and chemical semiquantitative analysis (by XRF-EDX) are reported in Table S1a and in Table S1b, respectively. The differences between Mica 10 and Mica 45 are mainly related to a different muscovite/kaolinite ratio.

The FTIR spectra (Figure S1), quite similar for both mica samples, agree with the mineralogic characterization achieved by XRD. The signals attributed to muscovite are present [51,52]. In the mid-IR region, the absorptions at 1113, 1065, and 1025 (Si-O), at 995 (Si-O-Si), at 911 (OH vibrational mode), at 829 (Al-O), at 751 (Al-O-Al), and at 693 (Si-O-Al_octahedral coord_) cm^−1^ are present. Of interest is also the region of OH stretching vibrations, where strong sharp absorptions at 3620 and 3693 cm^−1^ can be observed, which could be attributed to weak hydrogen bonds in a structure containing some impurities (2 Mg and 1 Al) with OH adjacent to an octahedral vacancy [51,52]. Another interesting feature from the FTIR spectra is the low water content in the mica samples, which is confirmed by the experimental results obtained upon heating the samples at 105 °C for 48 h (0.15% and 0.12% for Mica 10 and Mica 45, respectively).

The coordination of Si has been investigated through ^29^Si magic-angle spinning solid-state NMR. The ^29^Si solid-state NMR spectrum of Mica 45 reported in Figure S2 showed a main resonance at −91.6 ppm, attributed to Q4(4Al) Si sites of kaolinite Al_2_Si_2_O_5_(OH)_4_, refs. [53,54] together with broad minor components up to −60 ppm. The muscovite components were comprised in the broad part of the signal in the range from −81 to −85 ppm [55,56]. Briefly, ^29^Si chemical shifts of albites and microclines, both observed as secondary phases by XRD, have been reported in the literature in the range from −92 to −104 ppm [57], possibly contributing to the high-field broadening of the main peak at −91.6 ppm. The resonance at −107.5 ppm can be attributed to quartz, being a chemical shift typical of Si atoms tetrahedrally coordinated through an oxygen atom to other 4 Q4 Si atoms [58].

**Figure 2:**
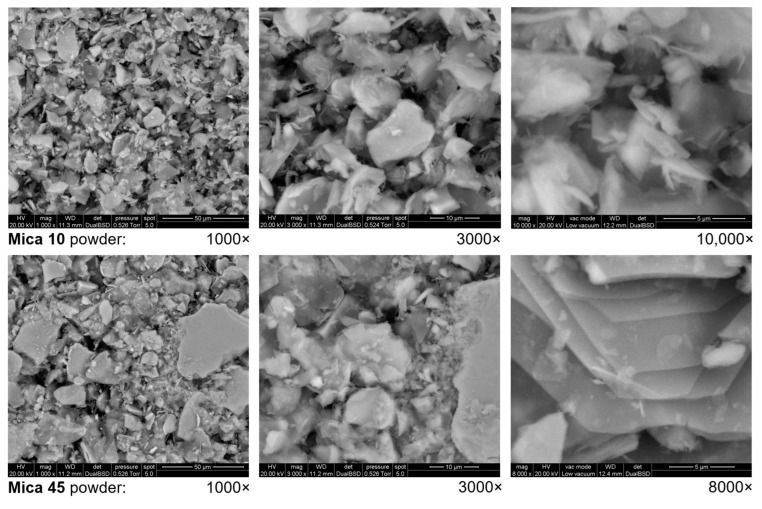
ESEM images of Mica 10 and Mica 45 at different magnifications.

A morphological characterization of the Mica 10 and Mica 45 powders has been carried out by ESEM. In Figure 2, it can be observed that the larger platelets are accompanied by the presence of smaller ones, as also results from the particle distribution, reported in Figure S3. From a large number of measurements carried out through ESEM, the aspect ratio in the range of 170–270 has been calculated.

It is noteworthy that the semiquantitative composition achieved by XRF determinations (reported in Table S2) confirmed the presence of Mg together with some other impurities of Fe and Ti, as observed for natural micas in relation to the metamorphic rocks from which they derive.

From these observations, we could conclude that the two mica samples have a quite similar composition, practically differing in the size distribution of particles and in the relative amounts of components, reasonably due to grinding and sieving processes. In the case of Mica 10, 56% of the volume is occupied by particles in the range of 8–14 μm, whereas in the case of Mica 45, 52% of the volume is occupied by particles in the range of 30–50 μm (Figure S3).

### 3.2. Chemical and Morphological Characterization of the Nanocomposites

**Figure 3:**
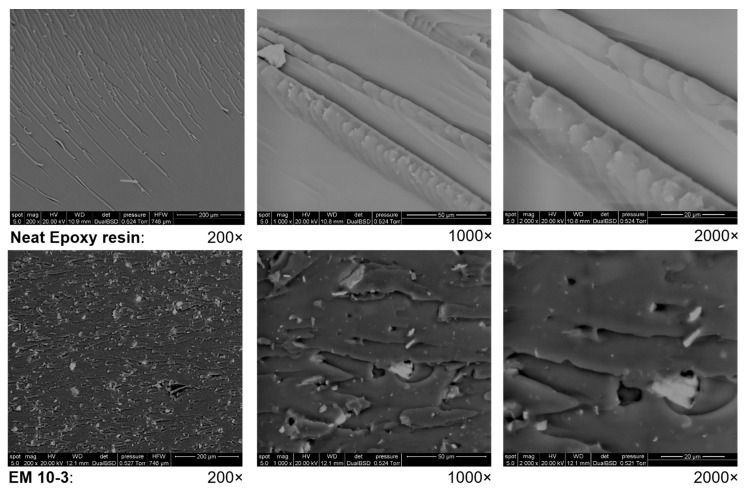
Representative ESEM images of the brittle fracture of the samples of the neat epoxy resin and composite EM10-3 at different magnifications.

According to the procedure described in the experimental section, the nanocomposites listed in Table 1 have been prepared. As shown by the ESEM images of the brittle fracture (Figure 3), a good dispersion has been achieved even if unmodified micas have been used, reasonably owing to the shape and dimension of the nanoparticles.

As shown in Figure 3 (see Figure S4 for the ESEM images of all the samples), the unfilled epoxy resin has a fairly smooth fracture surface, which is a typical brittle fracture for epoxy, suggesting that the material has low crack propagation resistance. The fillers act as a reinforcement for the epoxy matrix, thus inducing an increase in the energy required to propagate the cracks. A relatively weak adhesion between the micas and the matrix after stress load in all the nanocomposites was present, as demonstrated by the presence of small cavities around individual particles, indicating the high mechanical energy dissipative capacity of the mica particles [59].

**Figure 4:**
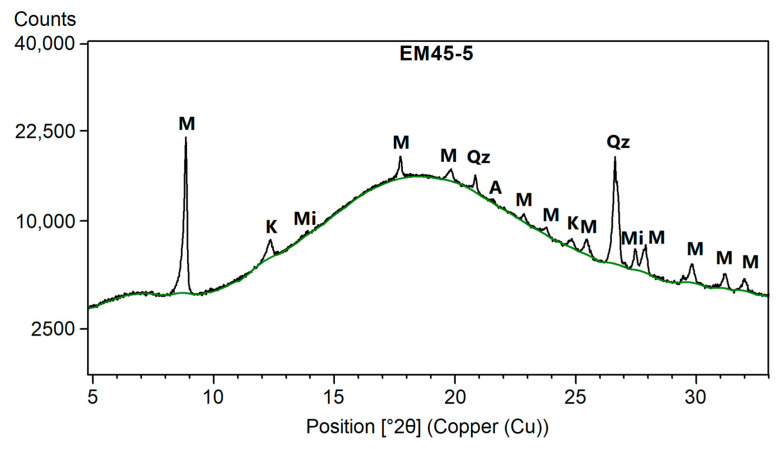
XRD of the EM45-5 sample (M = muscovite, K = kaolinite, Mi = microcline, A = albite, Qz = quartz).

In Figure 4 the XRD spectrum of the EM45-5 sample has been reported: it shows the signals corresponding to the mica filler superimposed to the signal of the epoxy resin, with no modification of the muscovite signals, indicating that the polymer was not able to enlarge the d_spacing_ of the mica.

**Figure 5:**
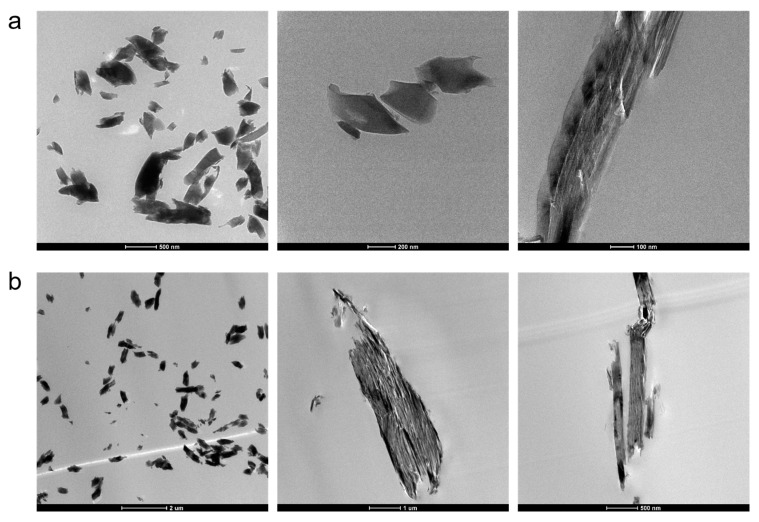
TEM micrographs of (**a**) the epoxy/mica nanocomposite EM10-5, and (**b**) the epoxy/mica nanocomposite EM45-5 at different magnifications.

TEM allows a qualitative observation of the internal structure of the nanocomposites through direct visualization of the filler distribution in the matrix. Figure 5 shows the TEM micrographs of the EM10-5 and EM45-5 nanocomposites; the nanoparticles are uniformly distributed within the epoxy matrix, and their dimensions in the matrix are similar to the initial dimensions of nanoparticles in the suspension, with some platelets alone even if few micro-aggregates were identified. The presence of the aggregates reduced the specific interface area thus being detrimental to the final nanocomposite properties.

**Figure 6:**
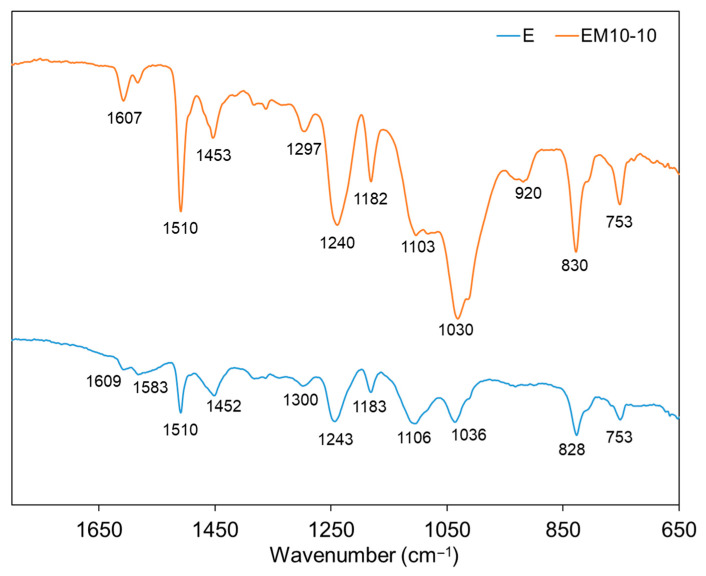
FTIR of the EM10-10 sample and of the neat epoxy resin (E).

In Figure 6, the FTIR spectra of the neat epoxy resin and of the EM10-10 sample have been reported in the range of 1900–600 cm^−1^. The characteristic absorptions of the pristine epoxy resin (ν_CCaromatic_, ν_CCOC_, ν_CN_, ν_COC_ ethers, and ν_COC_ oxirane at 1510, 1243, 1106, 10,362, and 827 cm^−1^, respectively) together with the strong absorption of Mica 10 at about 1000 cm^−1^ have been observed.

### 3.3. Thermal Behavior of the Nanocomposites

**Figure 7:**
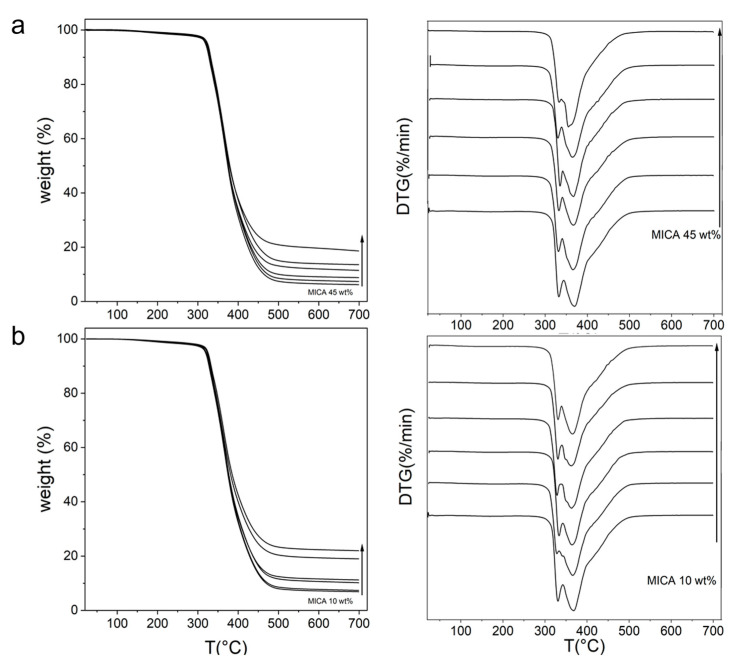
TGA (**left**) and DTG (**right**) curves for epoxy–mica (spanning from 0% wt to 15% wt) nanocomposites from RT to 700 °C under N_2_ atmosphere: (**a**) Mica 45 nanocomposites; (**b**) Mica 10 nanocomposites.

The thermal stability of all the samples was evaluated by TGA/DTG analysis. The thermograms and related time-derivative curves (DTG) are shown in Figure 7 for both the Mica 45- and Mica 10-filled epoxy matrices.

Neat epoxy material in the RT-250 °C range showed a loss of 1.73% due to the presence of water and volatiles, detected also for both Mica 45 and Mica 10 composites (1.20–1.86% wt). Neat epoxide decomposition started at an onset temperature of 325.2 °C with two peaks at 331.0 °C and 367 °C in the DTG profile, indicating at least a three-step process, considering the broadening of the DTG signal at T ≈ 400 °C. Table 2 summarizes the TGA and DTG parameters for assessing the effect of mica on the thermal stability of the composites, the onset temperature, the DTG peak temperature, and the temperature at which 5% weight loss occurred together with the residue at 700 °C.

The data reported in Table 2 and Figure S5 show that the parameters were not significantly affected by the introduction of mica fillers, in agreement with the data reported in the literature [60,61], where mica was less active compared with silica and CaCO_3_ toward thermal decomposition. As for residue values at 700 °C (Figure S6), a linear positive trend was found within the composition range for both Mica 10 and Mica 45 nanocomposites, in agreement with the consideration that by increasing the amount of inorganic filler, the final residue made of char and filler must increase. However, it was observed that for EM10-10 and EM10-15, the residual mass at 700 °C (under N_2_) was remarkably higher compared with the corresponding values for EM45-10 and EM45-15. This fact can be an indication of a more char-rich residue originating from the protection of the char formed during the thermal decomposition by the silicate layer, which, in turn, prevented char from further decomposing into more volatile compounds.

### 3.4. Optical Properties of the Nanocomposites

**Figure 8:**
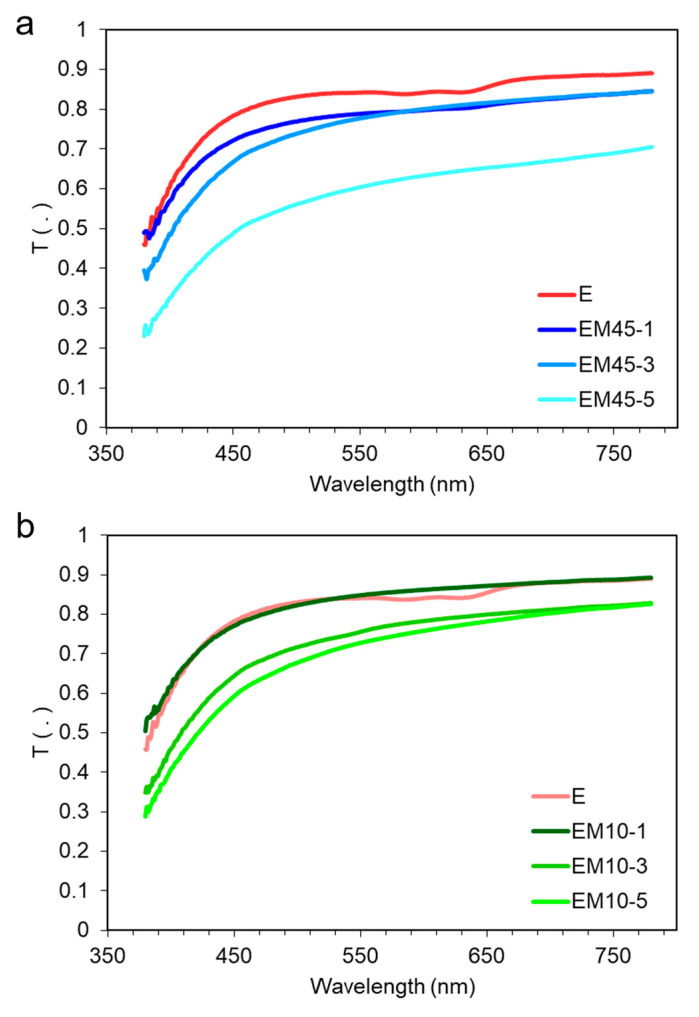
Measured spectral transmittance of (**a**) the samples with different fractions of Mica 10 and (**b**) the samples with different fractions of Mica 45.

The optical measurements referred only to the evaluation of the spectral transmittance in the visible domain of the samples containing 1, 3, and 5% wt of the micas, the thickness of which was 3.5 mm. The spectral transmittance measurements were obtained as the ratio of two spectral radiance measurements: the denominator is the output spectral radiance at the aperture of a reference radiance source, whereas the numerator is the fraction of that radiance after the light has passed through the sample. In Figure 8, the spectral transmittance functions of the nanocomposites have been reported compared with the transmittance of the neat epoxy resin. In all cases, a smoother behavior of the nanocomposites with respect to the neat sample was observed. It means a more neutral appearance of the nanocomposites, whereas the neat samples were more reddish due to the smooth step variation starting at about 650 nm. In Table S3, the values of the transmittance averaged over the considered wavelength range have been reported. It should be noted that in the case of nanocomposites containing 1% wt and 3% wt of mica fillers, the loss of transparency was lower than 10%. Comparing the effect of the two nanomicas, it appeared that 1% of Mica 45 did not significantly affect the average transmittance, whereas 1% of Mica 10 reduced the average transmittance by 5%. The reductions in the average transmittance due to the amount of 3% were comparable for the two nanomicas, and the difference was only 2%. A further increase in the mica amounts up to 5% produced a higher reduction in the average transmittance for Mica 10 (−39%). On the contrary, by increasing the Mica 45 amount from 3% to 5%, the average transmittance decreased only by 3%. The shape of the spectral transmittance for all nanocomposites did not significantly change. Increasing the mica amounts caused a reduction in the average transmittance, but also a small decline in the transmittance at the short wavelength with respect to the longest, thus shifting the appearance of the nanocomposites towards reddish, the variation being more evident at the highest amounts of Mica 10. The results agree with the attribution of the loss in transparency in the visible domain to the scattering of light by particles, which is closely linked to particle dimensions and the difference in refractive index between the particles and the medium [62]. The presence of micro-aggregates in the EM 10-5 sample could explain the reduced transmittance.

### 3.5. Resistive Properties of the Nanocomposites

Epoxy resin and mica are both good electrical insulating materials, and to characterize the electrical behavior of the mica-nanocomposites, the volume resistivity was evaluated. Before each measurement, the specimens were brought into electrically stable conditions by short-circuiting the measuring electrodes; to verify the repeatability of the results, more series of measurements were taken without significant differences. The results obtained from the measurements carried out according to the procedures reported in Section 3.3 have been summarized in Table 3.

The nanocomposite exhibited high values of volume resistivity, albeit lower compared with the epoxy nanocomposites containing hydrophobic nanosilica in the matrix (SiO_2_ 10% wt; resistivity from 3.2 × 10^15^ to 2.7 × 10^16^ Ω m) [63]. In any case, the values indicate that the composites are excellent insulating materials. It was observed that similar values have been obtained either in the case of Mica 10 and Mica 45 as nanofillers. A small influence on the volume resistivity seems related to the amount of mica and possibly due to a different effect on the space charge suppression, the limitation of the motions of molecular chains, and the prevention of the migration of charge carriers. Further experiments will be carried out to explore the dielectric behavior of the mica nanocomposites as a function of compositional parameters, epoxy-based dielectrics having a wide variety of applications in power equipment and electronic devices [64].

### 3.6. Mechanical Properties of the Nanocomposites

**Figure 9:**
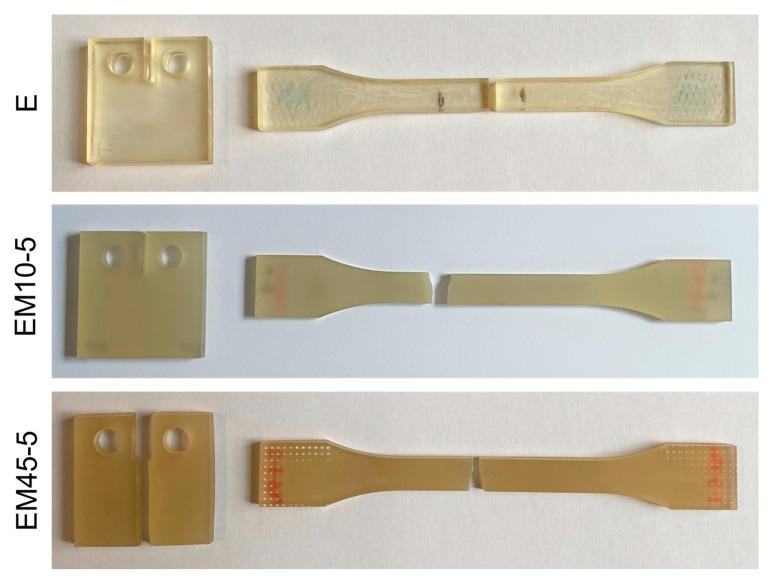
Selected specimens for fracture tests (CT) and tensile tests (DB) of neat epoxy resin (E), nanocomposite with 5% wt of Mica 10 (EM10-5), and nanocomposite with 5% wt of Mica 45 (EM45-5).

Tensile tests were conducted on dog-bone (DB) specimens according to the ISO 527-2 indications [44], and compact tension (CT) specimens complying with ASTM D5045-99 suggestions were used for the fracture tests [45]. Selected specimens are shown in Figure 9 for the neat epoxy resin and two nanocomposites with 5% wt of the two types of nanomicas.

The effect of the weight content of nanoclay on the nanocomposite tensile properties is shown in Table 4 in terms of average values, standard deviations, and variation with respect to the neat epoxy. Regardless of the filler type, the addition of nanoclay resulted in an increased Young’s modulus, roughly proportional to the filler amount. Compared to the neat polymer characterized by a Young’s modulus of 2.75 ± 0.05 GPa, the highest overall increase in the elastic modulus was reported for Mica 10 at 15% wt, with 3.64 ± 0.30 GPa (+32% increment). The average standard deviation to Young’s modulus ratio is about 6%, supporting the statistical soundness of the discussed trend. In contrast to the case of Young’s modulus, the nanocomposite tensile strength was negatively affected by the addition of nanoclay. The higher the filler content, the lower the tensile strength. Compared to the neat polymer characterized by a value of 66.2 ± 0.7 MPa, the lowest absolute value was that of Mica 45 at 5% wt, with a tensile strength of 52.4 ± 3.9 MPa (−21% increment). Again, the average standard deviation to tensile strength ratio was about 5%, supporting the statistical soundness of the discussed trend. Regarding the material fracture toughness, evaluated in terms of *K_Ic_* and reported in Table 5, nanomodification always resulted in a property enhancement. With respect to the fracture toughness of the neat epoxy (0.89 ± 0.13 MPa m^0.5^), the highest property enhancement was reported for Mica 10 at 15% wt, with a value of 1.47 ± 0.11 MPa m^0.5^ (+65% increment). The highest relative increase belonged to the lowest filler amount for all filler types, and Mica 45 had about a +35% average fracture toughness increase at 1% wt (1.21 ± 0.27 MPa m^0.5^). Even for this property, the average standard deviation-to-fracture toughness ratio was about 10%, supporting the statistical soundness of the discussed trend.

According to linear elasticity theory, a quasi-isotropic, quasi-homogeneous, multiphase material is characterized by elastic properties bounded between an upper and a lower limit, which depend only on each phase’s elastic properties and volume fraction [65]. It follows that processing able to disperse and distribute reinforcements homogeneously should guarantee a stable enhancement of the material Young’s modulus at increasing filler amounts as in the case of the samples here reported, as was proved by the ESEM images. Young’s modulus trend increased linearly with increasing filler amounts, and only Mica 10 at 15% wt showed a reduced effect of nanomodification. The value of the material’s Young’s modulus is statistically consistent across the different filler types when compared at the same filler amount, and it is also consistent with other values reported in the literature for similar material systems [66].

Concerning the material tensile strength, its trend is monotonic, even though it is not as consistently proportional as in the case of the material’s Young’s modulus. It is evident that nanomodification negatively affects the nanocomposite tensile strength, with a lower strength associated with higher filler content. Again, this trend is common to other similar material systems found in the literature [66,67] and it is of great importance when designing a composite nanomodification, given the trade-off between strength on one side and stiffness and fracture toughness on the other [67].

The improvement in fracture toughness is expected due to damage mechanisms that take place at the micro- and nano-scale following nanoplatelet addition [68]. In fact, nanoplatelets increase the roughness of the fracture surface, which increases the overall fracture surface area. Moreover, platelets’ tactoids and micro-aggregates are fully capable of interacting with the crack front, promoting toughening mechanisms such as crack pinning, crack deflection, and matrix deformation. Global trends show a progressive reduction in the effectiveness of clay addition. This condition is consistent with evidence reported in the literature, where the weakening of the overall mechanical properties of nanocomposite material systems, which can be observed when an optimum filler amount is exceeded, is discussed. It is in fact shown that, for each nanocomposite material system, once the saturation point of the nanofiller content is reached, the addition of higher amounts of reinforcing elements causes the deterioration of the overall mechanical properties of the material [68].

## 4. Numerical Modeling of Nanocomposite Mechanical Properties: Multiscale Peridynamic Approach

The multiscale peridynamic framework recently proposed in [38] is exploited here to model the effective Young’s modulus and fracture toughness of nanocomposite materials.

In the first part of the section, the RVE homogenization implemented to model the effective Young’s modulus of the nanomodified epoxy resins is briefly outlined, with a focus on the main phases of the modeling procedure and on the calibration of the numerical approach.

The second part of the section is instead dedicated to the discussion of the CCM-PD coupling strategy introduced in [39,40] and exploited here to model the fracture toughness of the epoxy resin nanocomposites. Even in this case, the modeling procedure and the calibration of the approach are briefly addressed.

As aforementioned, both numerical strategies are calibrated by exploiting the experimental data obtained from the tensile and fracture tests outlined in Section 3.6.

In order to better introduce the main features of the proposed multiscale PD framework, it is necessary to provide a brief outline of the PD theory. More details can be found in the literature [34,36] where the bond-based version of the theory and the more general state-based version of the theory are presented.

It should be noted that the calibration of the PD-based methods has been performed by considering only the experimental results obtained for the epoxy resins nanomodified with Mica 10, even though the same procedure can be easily employed to model the stiffness of the Mica 45-loaded resins. Given that the only difference between the two types of nanocomposites tested in this work lies in the characteristic size of the nanofillers and that the PD-based framework is not capable of accurately capturing the size effects [38], the computation of the Young’s modulus of the Mica 45-loaded samples is not of great interest and makes no significant contribution to the work presented here. The Mica 10 case study has been chosen because of the greater presence of data with respect to the Mica 45 case and thus the possibility of highlighting the flattening or drop in the mechanical performance that is observed when an optimum filler amount is exceeded.

The implementation of innovative PD-based approaches, such as micropolar PD models, to study the effects of the characteristic size of the nanofillers on the tensile and fracture properties of nanomodified epoxy resins is foreseen for the future.

### 4.1. Fundamentals of Peridynamics

In a domain B⊂R^n^ with n the spatial dimension, modeled by PD, each material point x∈B is associated with an infinitesimal volume d**V_x_** and interacts with all the other material points located within a finite neighborhood, $H_{x}$, of that material point. The bond-based PD equation of motion for any material point x∈B at time *t* ≥ 0 is given by the following integrodifferential equation:

(1) $$\rho \left(x\right)\ddot{u}\left(x,t\right)=\int_{H_{x}}f(u\left(x^{\prime },\;t\right)-u\left(x,\;t\right),\;\;\;x^{\prime }-x)dV_{x^{\prime }}+b\left(x,t\right),\;\;x^{\prime }\;\in \;H_{x},$$

where 𝜌 is the mass density, $\ddot{u}$ is the second derivative in time of the displacement field u, **f** denotes the pairwise force function that the material point $x^{\prime }$ exerts on the material point x, and **b** is a prescribed body force density field. The neighborhood $H_{x}:=\left\{\;x^{\prime }\in B:\;\|x^{\prime }-x\|\leq \delta \right\}$ is the integration region usually taken to be a line segment in 1D, a disk in 2D, and a sphere in 3D centered at x. The radius 𝛿 of $H_{x}\;$is called the horizon of x. The relative position vector between two material points *𝛏*:=$\;\;x^{\prime }-x$ is referred to as the bond. In the deformed configuration at time *t* > 0, the material points 𝐱 and 𝐱^′ ^would be displaced, respectively, by $u\left(x,\;t\right)\;$and $u\left(x^{\prime },\;t\right)$. The relative displacement vector is defined as $\eta :=u\left(x^{\prime },\;t\right)-u\left(x,\;t\right)$. The Prototype Microelastic Brittle (PMB) model for a linear elastic material is introduced, so that f, for the case of small deformations, is determined by [69]:

(2) $$f\left(u^{\prime }-u,\;x^{\prime }-x,t\right)=\mu \left(\xi ,t\right)\frac{c\omega \left(\xi \right)}{\left\|\xi \right\|}\|u\left(x^{\prime }\right)-u\left(x\right)\|e=\mu \left(\xi ,t\right)c\left(\left\|\xi \right\|\right)se,$$

where 𝜇 is a history-dependent damage function that, based on the bond status, takes either the value of 0 (broken bond) or 1 (active bond); $c\left(\left\|\xi \right\|\right)$ is the micromodulus function, *c* is the micromodulus constant, 𝜔 is the influence function that specifies the degree of nonlocal interactions between points, s is the relative elongation of a bond defined as s=(‖ξ+η‖−‖ξ‖)/‖ξ‖, **e** is the unit vector along the direction of the relative position vector in the current configuration; *c* is expressed in terms of material classical constants, i.e., *E* (Young’s modulus) and ν (Poisson’s ratio) [34]. In the PMB material model, the failure of a bond happens when s exceeds a predefined value $s_{0}$ which is related to the critical energy release rate of the material, *G*_0_ [69].

In this work, the meshfree discretization scheme is implemented [69]. The domain is discretized into nodes, each of them associated with a certain volume. Each node $x_{i}$ interacts with all nodes located within its neighborhood $H_{x_{i}}$*,* where $x_{i}$ is the source node and all $x_{j}$ are its family nodes. The horizon 𝛿 is expressed as 𝛿 = 𝑚 Δ𝑥, where 𝑚 is the ratio between 𝛿 and the grid spacing Δ𝑥. The spatial integration is pursued by adopting the one-point Gauss quadrature rule, and thus, the discretized form of (1) can be written as:

(3) $$\rho {\ddot{u}}_{i}^{n}=\underset{j}{\sum }f\left(u_{j}^{n}-u_{i}^{n},x_{j}-x_{i}\right)\beta \left(\xi \right)V_{j}+b_{i}^{n},\;\;\forall \;x_{j}\in H_{x_{i}},$$

where n is the time step, and subscripts denote the node number (e.g., $u_{j}^{n}$ = 𝐮(𝐱_𝑗_, 𝑡_𝑛_)); $\beta \left(\xi \right)$ is a partial-volume correction factor, as introduced in [70]. In this work, a uniform grid with Δ𝑥 = Δ𝑦, where Δ𝑥 and Δ𝑦 are the grid spacings in the 𝑥- and 𝑦-directions, respectively, is considered.

### 4.2. PD-BASED RVE Approach: Modeling of Effective Young’s Modulus

#### 4.2.1. Modeling Strategy

The nanocomposite Young’s modulus is derived by implementing a PD-based RVE homogenization, whose results are then employed in the fracture analysis performed in the second part of the section [38].

The modeling procedure is subdivided into different phases, i.e., the determination of constituents’ properties, the selection and modeling of a suitable RVE, the performance of the static analysis, and the computation of the elastic constants to obtain the effective material properties. The first phase consists of the definition of the mechanical and geometrical properties of both the nanofiller and the matrix materials. In this work, the required constituents’ properties have been extrapolated from the testing campaign described in Section 3.6 and from data available in literature studies. Nanofiller size, orientation, and position are considered to be affected by uncertainties and modeled by identifying suitable probability distribution functions [38]. These properties are then used as input for the mesoscale analysis, which is performed on an RVE, a sample that is structurally typical of the whole blend on average. The nanocomposites’ properties are computed by implementing several RVEs and by aggregating the results. Moreover, the required number of material realizations to obtain suitable Young’s modulus values should be determined. As comprehensively outlined in [71], a sufficient number of inclusions should also be contained within the RVE domain, so that the overall moduli can be independent of the surface values of traction and displacement. The random distribution of nanofillers is modeled by assigning different material properties to the PD nodes employed to discretize the square RVE domains. A newly developed algorithm allows to model nanofiller curvature, which is an often-overlooked aspect, even if previous studies have proved that nanoclays dispersed within a polymer blend do not preserve their straight shape due to their high aspect ratio [72,73]. A random size and orientation are automatically assigned to the nanofillers during the modeling procedure. The domain is modeled as geometrically periodic, i.e., the nanofillers that are cut by any of the edges of the RVE are continued from the opposite edges with the same orientation and curvature [38]. The properties assigned to each bond depend on the nature of the nodes at its ends. Matrix–matrix, nanofiller–nanofiller, and matrix–nanofiller (or interface) bonds are therefore defined. The stiffness of matrix–matrix and nanofiller–nanofiller bonds is modeled employing the Young’s modulus of the matrix and that of the nanofiller, i.e., *E_m_* and *E_nf_*, respectively. The Young’s modulus of the matrix–nanofiller bonds is instead modeled as $E_{int}=\kappa_{int}E_{m}$, where *κ_int_* is referred to as interface factor. In this study, the experimental results reported in Section 3.6 are exploited to calibrate the interface factor. The stiffness value assigned to the agglomeration bonds, i.e., nanofiller–nanofiller bonds connecting nanofiller-type nodes belonging to different nanoplatelets, is the lower between the matrix and interface Young’s moduli, depending on the case study [38].

The next step of the modeling procedure is the implementation of the static analysis. After the assembly of the global RVE stiffness matrix, the boundary conditions are imposed on a volume of boundary layers surrounding the RVE domain with a depth equaling the horizon. A uniform strain condition is applied to the RVE by imposing on all the nodes in the external boundary layers a displacement which is a linear approximation of the boundary displacement. In this way, it is possible to eliminate the so-called surface effect (see [38]). The computed reaction forces are then processed to evaluate the material elastic constants and therefore to obtain the effective Young’s modulus of the nanomodified epoxy resins [38].

#### 4.2.2. Numerical Model Calibration

As comprehensively discussed in [38], before proceeding with the calibration procedure, the minimum RVE size and the required number of RVE realizations need to be determined. An RVE side length of *L_RVE_* = 50 μm is considered [38], and each case is averaged over 100 RVE realizations. Table 6 reports the input data employed in the calibration of the RVE-based approach. The values of *E_nf_*, *ρ_nf_*, AR_mean_, and AR_std_ (i.e., the mean value and standard deviation of the nanomica’s aspect ratio) are reported in available experimental investigations and technical reports [74], whereas the values of *ν_m_* and *ν_nf_* are fixed for plane stress conditions [34,75]. Uniaxial strain conditions are imposed as described in [38].

**Figure 10:**
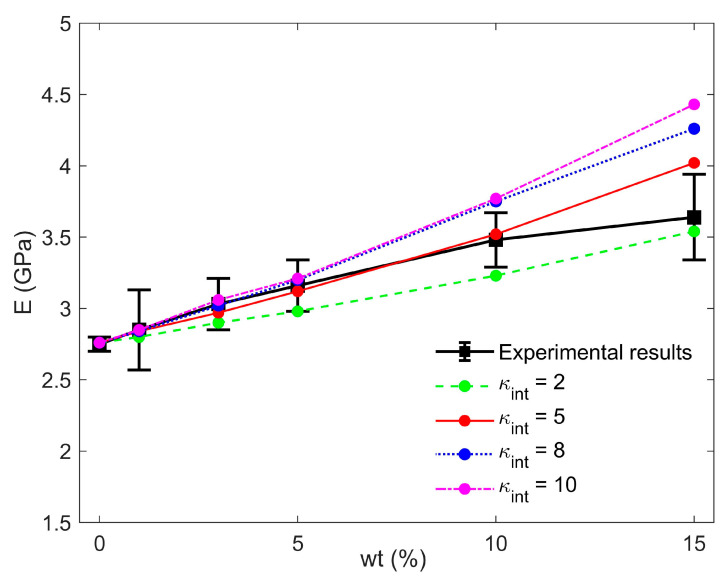
Results of the PD-based RVE approach calibration.

In the calibration procedure, the same nanofiller contents employed in the experimental investigation reported in Section 3.6 are considered, i.e., 0%, 1%, 3%, 5%, 10%, and 15% wt of the Mica 10 amount. The Young’s modulus value experimentally obtained for the samples with 5% wt of clay amount is exploited for model calibration purposes. Figure 10 illustrates the obtained results. It is demonstrated that, for *κ_int_* = 5, the model is capable of predicting the average Young’s modulus value for all the nanocomposite configurations with a maximum error of less than 3.5%. The only result that slightly differs from that obtained from the experimental tests is the 15% wt one, with an error of about 8%. This discrepancy could be related to the criterion used to define the stiffness of the agglomeration bonds (see Section 4.2.1), which may not be very effective in simulating the presence of very large agglomerates within the matrix in cases in which the amount of nanofillers exceeds a certain optimum value, after which, the mechanical properties begin to deteriorate (see Table 4). A more in-depth study of this aspect is foreseen for the future.

The effective Young’s modulus values obtained through the calibration procedure are exploited in the fracture analysis outlined in Section 4.3.

### 4.3. CCM-PD Coupling Approach: Modeling of Fracture Toughness

#### 4.3.1. Modeling Strategy

The fracture toughness of the epoxy resin nanocomposites is derived by exploiting the CCM-PD coupling strategy [39,40]. The nanocomposites fracture toughness is studied by modeling a simplified standard CT specimen [38,45], in which the region affected by pre-crack propagation is described by employing the intermediately homogenized peridynamic (IH-PD) model introduced in [76], whereas the remaining part of the model domain is described using the finite element method (FEM). As for the description of the FEM region, which is modeled as fully homogeneous, the effective Young’s moduli obtained through the RVE approach outlined in Section 4.2 are exploited for modeling the mechanical properties of the FEM elements. In the PD region, the mechanical properties of the bonds are instead modeled by exploiting the stochastic procedure outlined in [76]. Three types of bonds are generated and stochastically distributed within the model domain, i.e., matrix–matrix, nanofiller–nanofiller, and matrix–nanofiller, or interface, bonds. The stiffness of matrix–matrix and nanofiller–nanofiller bonds is modeled employing the Young’s modulus of the matrix and that of the nanofiller, i.e., *E_m_* and *E_nf_*, respectively. The Young’s modulus of the matrix–nanofiller bonds is instead modeled employing the harmonic averaging method [38,76].

In order to compute the critical stretches of the different bonds generated by the stochastic procedure, the critical energy release rate values are defined as follows: the critical energy release rate of the matrix, i.e., $G_{0_{m}}$, is exploited to model the matrix–matrix bonds, whereas $G_{0_{nf}}$ and $G_{0_{int}}$ are employed to model the nanofiller–nanofiller and matrix–nanofiller bonds, respectively, and are modeled as a function of $G_{0_{m}}$, such that [38]:

(4) $$G_{0_{nf}}=G_{0_{int}}=\chi G_{0_{m}},$$

where χ is referred to as the fracture energy factor. In this work, the experimental results presented in Section 3.6 are exploited to calibrate this factor. More details can be found in [38], where the derivation of this modeling criterium (cf. (4)) is outlined.

The determination of the mechanical properties to be allocated to the different types of bonds is followed by the implementation of an improved version of the stochastic procedure presented in [76], where the proposed algorithm is modified to consider the coupling zone, i.e., the region where forces are exchanged between the FEM and PD parts of the domain. This region is therefore characterized by the presence of bonds whose properties are the ones obtained through the RVE-based model presented in Section 4.2.

The last phase of the modeling procedure is the implementation of the quasi-static fracture analysis, as comprehensively outlined in [38,77]. The proposed CCM-PD configuration enables the use of classical local boundary conditions, thus avoiding the need for external boundary layers, as was the case with the RVE-based approach presented in Section 4.2. The structural problem is therefore solved by implementing a sequentially linear analysis (SLA) [77]. The sum of the reaction forces at the nodes where the prescribed displacements are enforced is then processed to obtain the fracture toughness of the nanomodified epoxy resins [38].

#### 4.3.2. Numerical Model Calibration

Table 7 reports the experimental data employed to calibrate the coupling-based strategy.

The fracture tests are simulated by modeling a simplified CT specimen, which is represented by a plate with an internal PD portion surrounded by the FEM region. The PD portion is a rectangle of edge lengths $L_{PD_{x}}$ = 9.88 mm and $L_{PD_{y}}$ = 6.68 mm, whose center has coordinates (2.4,0) mm. The values of the main problem parameters are *L_x_* = 40.4 mm and *L_y_* = 38.8 mm (plate dimensions) and *h* = 5 mm (plate thickness). The model domain is discretized using a uniform grid, i.e., ∆*x* = ∆*y*, both for the PD and FEM regions. In all simulations, the initial pre-crack length is considered to be equal to a = 13 mm. The values of *E_m_*, *E_nf_*, *ρ_m_*, and *ρ_nf_* have been collected from the characterization carried out in Section 3.6 and are also reported in available experimental investigations and technical reports [74], whereas the values of *ν_m_* and *ν_nf_* are fixed for plane strain conditions [34,75]. The value assigned to $G_{0_{m}}$ has been evaluated through the following relation [38]:

(5) $$G_{0_{m}}=\frac{K_{Ic_{m}}{}^{2}}{E_{m}}\left(1-\nu_{DGEBA}{}^{2}\right),$$

where $K_{Ic_{m}}$ is the experimentally obtained fracture toughness value for the neat epoxy resin (see Section 3.6), *E_m_* represents the experimentally obtained Young’s modulus value for the neat epoxy (see Section 3.6), and *ν_DGEBA_* is the matrix Poisson’s ratio, which is considered equal to 0.35 [46,73]. The relation in (5) is valid under plane strain conditions [45]. As aforementioned, the value of $E_{int}$ is instead evaluated by exploiting the harmonic averaging method (see [38]), whereas the effective Young’s moduli obtained through the RVE homogenization procedure presented in Section 4.2 are exploited for the modeling of the properties of both the FEM region and the coupling zone.

An upward vertical displacement of *u_y_* = 1 μm and a downward vertical displacement of *u_y_* = −1 μm are imposed on the FEM node at coordinates (−12.27, 9.33) mm and on the FEM node at coordinates (−12.27, −9.33) mm, respectively. Moreover, the two aforementioned FEM nodes are not allowed to move in the horizontal direction, i.e., *u_x_* = 0 μm. The SLA procedure recalled in Section 4.3.1 is exploited to compute the nodal reaction forces of the system, which are then employed to evaluate the fracture toughness of the nanocomposites by means of the following relation [45]:

(6) $$K_{Ic}=\frac{P_{\mathrm{cr}}}{{\mathrm{BW}}^{0.5}}f\left(x\right),$$

where P_cr_ is the fracture load (or peak value of the reaction forces), B is the thickness of the specimen, W is referred to as the ligament, x = a/W represents the ratio between the initial pre-crack length and the ligament, and f(x) is expressed as:

(7) $$f\left(x\right)=\frac{\left(2+x\right)\left(0.886+4.64x-13.32x^{2}+14.72x^{3}-5.6x^{4}\right)}{{\left(1-x\right)}^{1.5}},$$

which is valid for 0.2 < x < 0.8.

In all simulations, B = ℎ and W = 32.40 mm. The same nanofiller contents considered in Section 3.6, i.e., 0%, 1%, 3%, 5%, 10%, and 15% wt of Mica 10, are also employed in the modeling procedure. To consider model stochasticity, each calculation point is averaged over three runs.

**Figure 11:**
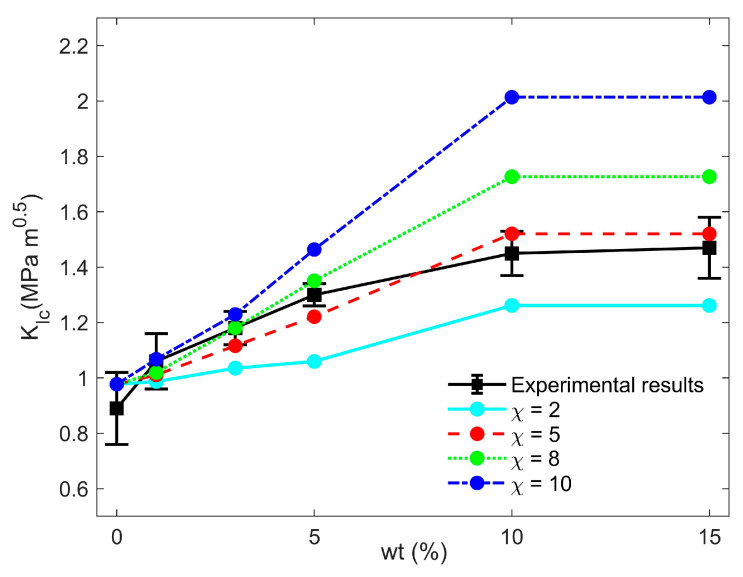
Results of the numerical model calibration.

The fracture toughness value experimentally obtained for the samples with 5% wt of clay amount is exploited for model calibration purposes. Figure 11 illustrates the obtained results. It is demonstrated that, for χ = 5, the model is capable of predicting the average value of the fracture toughness for all the nanocomposite configurations. As demonstrated by the results shown in Table 5, after exceeding a clay amount of 10% wt, *K_Ic_* reached a plateau. As a direct consequence, the fracture toughness for the 15% wt case is considered here equal to the one numerically obtained for the 10% wt case. As comprehensively discussed in [38], this behavior may be caused by the coalescence of large nanoclay agglomerates, which hinders a further increment of the fracture toughness value. More in-depth studies of this aspect and the consequent extension of the proposed approach to the modeling of nanofiller agglomeration phenomena and their effects on the fracture toughness of nanocomposites are foreseen for the future.

## 5. Conclusions

The preparation of epoxy nanocomposites containing two natural micas, Mica 10 and Mica 45, which have been characterized by XRD and ESEM, has been described. Due to their nanometric particle size distribution and high aspect ratio (higher than 170), a homogeneous dispersion was achieved even without being organically modified. This imparted reduced transmittance properties of less than 10% in the spectral range of 350–800 nm to the final materials with up to 5% wt filler content, thus yielding unexpected transparent nanocomposites.

The lack of intercalation and exfoliation can explain the small differences in the thermal behavior and mechanical properties of the epoxy resin composites despite the difference in the particle size distribution of the two micas. Their addition to the epoxy matrix resulted in an increased Young’s modulus, whereas tensile strength decreased. Both effects were enhanced by the increased amount of micas present. The fracture toughness was increased by the presence on the nanofillers by 65% with respect to the unfilled epoxy resin in the case of mica 10 at 15% wt/wt, whereas for mica 45, an increase of 35% was observed already at 1% wt/wt. These can be explained by the damage mechanism taking place at the micro and nanoscale as a consequence of the presence of nanoplatelets, which increase the roughness and area of the fracture surface. The data obtained in the tensile and fracture tests have been studied also exploiting peridynamics-based approaches, which properly reproduced the experimental results and allowed to explain the trends in terms of the morphological structure of the nanocomposites.

Both Mica 10- and Mica 45-containing nanocomposites exhibited high values of volume resistivity, thus being excellent insulating materials.

The results of the study invite further investigations, in particular, a more in-depth study of the morphology of nanocomposites and matrix–nanofiller interaction, specifically for non-organically modified nanoclays, with the aim of designing and manufacturing multifunctional and increasingly performing nanocomposites, such as transparent materials for packaging.

## Figures and Tables

**Table 1:** Experimental details for nanocomposite preparation and labeling.

	Mica Amount (g) (% wt/wt)	Sample Labeling	Density (g/cm^3^) ^a^
Epoxy resin	0 g (0%)	E	1.157
Mica 10	1.6 g (1%)	EM10-1	1.161
	4.8 g (3%)	EM10-3	1.179
	8.0 g (5%)	EM10-5	1.193
	16.0 g (10%)	EM10-10	1.224
	24.0 g (15%)	EM10-15	1.251
Mica 45	1.6 g (1%)	EM45-1	1.162
	4.8 g (3%)	EM45-3	1.175
	8.0 g (5%)	EM45-5	1.190
	16.0 g (10%)	EM45-10	1.224
	24.0 g (15%)	EM45-15	1.252

^a^ The density determinations were performed with an XPR/XSR analytical density kit (Mettler Toledo). The data are the average of three independent measurements.

**Table 2:** Parameters from thermal analysis of pristine epoxy and mica-composites in N_2_.

Sample	T °C Onset	T °C @5% wt Loss	% Residual mass@699 °C	T °C I dec	T °C II dec
E	325.2	322.5	6.48	331.0	367.0
EM10-1	323.6	318.8	8.44	328.3	365.8
EM10-3	326.5	324.8	10.31	333.3	364.1
EM10-5	322.9	320.2	11.33	328.6	363.1
EM10-10	326.3	325.1	22.27	330.0	363.2
EM10-15	323.8	323.1	17.53	330.7	362.4
EM45-1	325.8	320.5	7.46	331.3	365.6
EM45-3	323.6	318.7	8.94	328.5	363.1
EM45-5	326.5	324.3	11.61	333.1	365.1
EM45-10	325.3	321.4	13.77	331.3	365.6
EM45-15	326.9	323.5	12.51	333.0	355.0

**Table 3:** Volume resistivity measured for the mica nanocomposites containing the highest amounts of mica fillers.

Sample	Volume Resistivity [Ω m × 10^12^]
EM10-5	28
EM45-5	33
EM10-10	18
EM45-10	22
EM10-15	9
EM45-15	13

**Table 4:** Results from tensile tests. Values are reported in terms of mean value and standard deviation. Variation column reports the variation of the mean value with respect to neat epoxy.

Sample	Young’s Modulus [GPa]	Young’s Modulus var [%]	Tensile Strength [MPa]	Tensile Strength var [%]
E	2.75 ± 0.05	-	66.2 ± 0.7	-
EM10-1	2.85 ± 0.28	4%	64.8 ± 3.2	−2%
EM10-3	3.03 ± 0.18	10%	63.6 ± 1.5	−4%
EM10-5	3.13 ± 0.26	14%	56.7 ± 2.8	−14%
EM10-10	3.48 ± 0.19	27%	60.4 ± 3.5	−9%
EM10-15	3.64 ± 0.30	32%	57.1 ± 6.1	−14%
EM45-1	2.88 ± 0.17	4%	61.7 ± 1.7	−7%
EM45-3	3.09 ± 0.24	12%	55.0 ± 3.4	−17%
EM45-5	3.28 ± 0.06	19%	52.4 ± 3.9	−21%

**Table 5:** Results from fracture tests. Values are reported in terms of mean value and standard deviation. Variation column reports the variation of the mean value with respect to neat epoxy.

Sample	Fracture Toughness *K_Ic_* [MPa m^0.5^]	Var [%]
E	0.89 ± 0.13	-
EM10-1	1.06 ± 0.10	19%
EM10-3	1.18 ± 0.06	33%
EM10-5	1.30 ± 0.04	46%
EM10-10	1.45 ± 0.08	63%
EM10-15	1.47 ± 0.11	65%
EM45-1	1.21 ± 0.27	36%
EM45-3	1.22 ± 0.11	37%
EM45-5	1.43 ± 0.09	61%

**Table 6:** Input data for the calibration procedure.

Input Data	Value
Δ𝑥 = Δ𝑦	250 nm
*m*	4
*E_m_*	2.75 GPa
*E_nf_*	190 GPa
*ρ_m_*	1156.5 kg/m^3^
*ρ_nf_*	2820 kg/m^3^
*ν_m_* = *ν_nf_*	1/3
AR_mean_	200
AR_std_	30

**Table 7:** Input data for the calibration procedure.

Input Data	Value
Δ𝑥 = Δ𝑦	80 μm
*m*	4
*E_m_*	2.75 GPa
*E_nf_*	190 GPa
*ρ_m_*	1156.5 kg/m^3^
*ρ_nf_*	2820 kg/m^3^
*ν_m_ = ν_nf_*	1/4
$G_{0_{m}}$	252.8 J/m^2^

## Data Availability

Data will be made available on request.

## Supplementary Materials

The following supporting information can be downloaded at: https://www.mdpi.com/article/10.3390/polym15061456/s1, Table S1: Mineralogical composition of micas from XRD data; Table S2: (a) Composition of Mica 10 by XRF data, (b) Composition of Mica 45 from XRF data; Table S3: Values of the average transmittance for the tested nanocomposites; Figure S1: FTIR spectra of Mica 10 and Mica 45 (a) in the 3000–4000 cm^−1^ region (b) and in 600–1250 cm^−1^ region; Figure S2: ^13^C solid-state NMR spectrum of Mica 45; Figure S3: Particle size distribution of Mica 10 and Mica 45 powders; Figure S4: ESEM images at different magnifications of the neat epoxy resin and the EM composites; Figure S5: T_onset_ decomposition as a function of nominal filler weight percentages for Mica 10 and Mica 45 nanocomposites; Figure S6: Residual mass (calculated from dry mass) at 700 °C as function of nominal filler weight percentages for Mica 10 (void circles) and Mica 45 (full circles) nanocomposites.

## References

[1] Paul D., Robeson L. (2008). Polymer nanotechnology: Nanocomposites. Polymer.

[2] Pavlidou S., Papaspyrides C.D. (2008). A review on polymer–layered silicate nanocomposites. Prog. Polym. Sci..

[3] Vimalathithan P.K., Barile C., Casavola C., Vijayakumar C.T., Arunachalam S., Battisti M.G., Friesenbichler W. (2018). Investigation on the Thermal Degradation Kinetics of Polypropylene/Organically Modified Montmorillonite Nanocomposites with Different Levels of Compatibilizer. Macromol. Mater. Eng..

[4] Tan L., He Y., Qu J. (2019). Structure and properties of Polylactide/Poly(butylene succinate)/Organically Modified Montmorillonite nanocomposites with high-efficiency intercalation and exfoliation effect manufactured via volume pulsating elongation flow. Polymer.

[5] Kausar A., Ahmad I., Maaza M., Eisa M. (2022). State-of-the-Art Nanoclay Reinforcement in Green Polymeric Nanocomposite: From Design to New Opportunities. Minerals.

[6] Pascault J.P., Williams R.J.J. (2010). Epoxy Polymers: New Materials and Innovations.

[7] Ogbonna V.E., Popoola A.P.I., Popoola O.M. (2022). A review on recent advances on the mechanical and conductivity properties of epoxy nanocomposites for industrial applications. Polym. Bull..

[8] Karak N. (2006). Polymer (epoxy) clay nanocomposites. J. Polym. Mat..

[9] Nanda T., Sharma G., Mehta R., Shelly D., Singh K. (2019). Mechanisms for enhanced impact strength of epoxy based nanocomposites reinforced with silicate platelets. Mater. Res. Express.

[10] Gul S., Kausar A., Muhammad B., Jabeen S. (2016). Technical Relevance of Epoxy/Clay Nanocomposite with Organically Modified Montmorillonite: A Review. Polym. Technol. Eng..

[11] Zabihi O., Ahmadi M., Nikafshar S., Preyeswary K.C., Naebe M. (2018). A technical review on epoxy-clay nanocomposites: Structure, properties, and their applications in fiber reinforced composites. Compos. Part B Eng..

[12] Ali A., Mohammed A.S., Merah N. (2018). Tribological Investigationd of UHMWPE Nanocomposites Reinforced with three Different Organo-Modified Clays. Polym. Compos..

[13] Shuai C., Yu L., Feng P., Zhong Y., Zhao Z., Chen Z., Yang W. (2020). Organic montmorillonite produced an interlayer locking effect in a polymer scaffold to enhance interfacial bonding. Mater. Chem. Front..

[14] Bischoff E., Simon D.A., Liberman S.A., Mauler R.S. (2020). Influence of the dispersing agents to obtain polymer–clay nanocomposites processed in two-steps using thermokinetic mixer. J. Mater. Sci..

[15] Andraschek N., Wanner A.J., Ebner C., Riess G. (2016). Mica/Epoxy-Composites in the Electrical Industry: Applications, Composites for Insulation, and Investigations on Failure Mechanisms for Prospective Optimizations. Polymers.

[16] Moskalyuk O.A., Belashov A.V., Beltukov Y.M., Ivan’Kova E.M., Popova E.N., Semenova I.V., Yelokhovsky V.Y., Yudin V.E. (2020). Polystyrene-Based Nanocomposites with Different Fillers: Fabrication and Mechanical Properties. Polymers.

[17] Fahami A., Lee J., Lazar S., Grunlan J.C. (2020). Mica-Based Multilayer Nanocoating as a Highly Effective Flame Retardant and Smoke Suppressant. ACS Appl. Mater. Interfaces.

[18] Pan X.-F., Gao H.-L., Wu K.-J., Chen S.-M., He T., Lu Y., Ni Y., Yu S.-H. (2020). Nacreous aramid-mica bulk materials with excellent mechanical properties and environmental stability. iScience.

[19] Jia F., Su J., Song S. (2015). Can natural muscovite be expanded?. Colloids Surf. A.

[20] Wang Y., Liu Q., Zhen Z., Liu J., Qiao R., He W. (2021). Effects of mica modification with ethylene-vinyl acetate wax on the water vapor barrier and mechanical properties of poly-(butylene adipate-co-terephthalate) nanocomposite films. J. Appl. Polym. Sci..

[21] Mohammadi H., Moghbeli M.R. (2019). Polypropylene/organically modified-grafted mica/organoclay hybrid nanocomposites: Preparation, characterization, and mechanical properties. Polym. Compos..

[22] Almeida L.A., Marques M.F.V., Dahmouche K. (2018). Synthesis, structure, and thermal properties of new polypropylene nanocomposites prepared by using MgCl_2_-mica/TiCl_4_ based catalyst. J. Appl. Polym. Sci..

[23] Ziadeh M., Fischer B., Schmid J., Altstädt V., Breu J. (2014). On the importance of specific interface area in clay nanocomposites of PMMA filled with synthetic nano-mica. Polymer.

[24] Huang W., Komarneni S., Gorski C., Noh Y.D., Doroski A., Dong Y., Ma J., Griffin A.M., Yang D., Xue X. (2020). Few-Layer Clayenes for Material and Environmental Applications. ACS Appl. Mater. Interfaces.

[25] Yazdani H., Morshedian J., Khonakdar H.A. (2006). Effects of silane coupling agent and maleic anhydride-grafted polypropylene on the morphology and viscoelastic properties of polypropylene–mica composites. Polym. Compos..

[26] Xiao C., Li D., Zeng D., Lang F., Xiang Y., Lin Y. (2020). A comparative investigation on different silane coupling agents modified sericite mica/polyimide composites prepared by in situ polymerization. Polym. Bull..

[27] Malyshev M.D., Guseva D.V., Vasilevskaya V.V., Komarov P.V. (2021). Effect of Nanoparticles Surface Bonding and Aspect Ratio on Mechanical Properties of Highly Cross-Linked Epoxy Nanocomposites: Mesoscopic Simulations. Materials.

[28] Uno H., Tamura K., Yamada H., Umeyama K., Hatta T., Moriyoshi Y. (2009). Preparation and mechanical properties of exfoliated mica-polyamide 6 nanocomposites using sericite mica. Appl. Clay Sci..

[29] Messersmith P.B., Giannellis E.P. (1994). Synthesis and Characterization of Layered Silicate-Epoxy Nanocomposites. Chem. Mater..

[30] Pothukuchi H.K.R., Fuchs P., Pinter G., Stelzer S. (2018). Fracture mechanical characterization of mica-filled epoxy glass composites under monotonic and cyclic loading. J. Compos. Mater..

[31] Mo H., Wang G., Liu F., Jiang P. (2016). The influence of the interface between mica and epoxy matrix on properties of epoxy-based dielectric materials with high thermal conductivity and low dielectric loss. RSC Adv..

[32] Boukerrou A., Duchet J., Fellahi S., Sautereau H. (2007). Processing of mica/epoxy nanocomposites by ultrasound mixing. J. Appl. Polym. Sci..

[33] Noll W. (1974). A new Mathematical Theory for Simple Materials. The Foundation of Mechanics and Thermodynamics.

[34] Silling S. (2000). Reformulation of elasticity theory for discontinuities and long-range forces. J. Mech. Phys. Solids.

[35] Silling S.A., Bobaru F., Foster J.T., Gaubelle P.H., Silling S.A. (2016). Why Peridynamics?. Handbook of Peridynamic Modeling.

[36] Silling S.A., Epton M., Weckner O., Xu J., Askari E. (2007). Peridynamic States and Constitutive Modeling. J. Elast..

[37] Shojaei A., Hermann A., Seleson P., Silling S.A., Rabczuk T., Cyron C.J. (2023). Peridynamic elastic waves in two-dimensional unbounded domains: Construction of nonlocal Dirichlet-type absorbing boundary conditions. Comput. Methods Appl. Mech. Eng..

[38] Ongaro G., Bertani R., Galvanetto U., Pontefisso A., Zaccariotto M. (2022). A multiscale peridynamic framework for modelling mechanical properties of polymer-based nanocomposites. Eng. Fract. Mech..

[39] Galvanetto U., Mudric T., Shojaei A., Zaccariotto M. (2016). An effective way to couple FEM meshes and Peridynamics grids for the solution of static equilibrium problems. Mech. Res. Commun..

[40] Zaccariotto M., Mudric T., Tomasi D., Shojaei A., Galvanetto U. (2018). Coupling of FEM meshes with Peridynamic grids. Comput. Methods Appl. Mech. Eng..

[41] Ongaro G., Seleson P., Galvanetto U., Ni T., Zaccariotto M. (2021). Overall equilibrium in the coupling of peridynamics and classical continuum mechanics. Comput. Methods Appl. Mech. Eng..

[42] Ongaro G. (2022). Simulation of Damage Propagation in Materials and Structures by Using Peridynamics. Ph.D. Thesis.

[43] Massiot D., Fayon F., Capron M., King I., Le Calvè S., Alonso B., Durand J.O., Bujoli B., Gan Z., Hoats G. (2002). Modelling One- and two-dimensinal solid-state NMR spectra. Magn. Reson. Chem..

[44] (2012). Plastics-Determination of Tensile Properties. Part 2: Test Conditions for Moulding and Extrusion Plastics.

[45] (1999). Standard Test Methods for Plane-Strain Fracture Toughness and Strain Energy Release Rate of Plastic Materials.

[46] Zappalorto M., Salviato M., Quaresimin M. (2013). Mixed mode (I + II) fracture toughness of polymer nanoclay nanocomposites. Eng. Fract. Mech..

[47] Fiorentin P., Scroccaro A. (2010). Detector-Based Calibration for Illuminance and Luminance Meters—Experimental Results. IEEE Trans. Instrum. Meas..

[48] (2016). Dielectric and Resistive Properties of Solid Insulating Materials. Part 3-1: Determination of Resistive Properties (DC Methods)—Volume resistance and volume resistivity—General Method.

[49] Ferraris G., Ivaldi G., Mottana A., Sassi F.P., Thompson J.B., Guggenheim S. (2002). Structural Features of Micas. Micas: Crystal Chemistry and Metamorphic Petrology.

[50] Guidotti C.V., Sassi F.P., Mottana A., Sassi F.P., Thompson J.B., Guggenheim S. (2002). Constrains on Studies of Metamorphic K-Na White Micas. Micas: Crystal Chemistry and Metamorphic Petrology.

[51] Beran A., Mottana A., Sassi F.P., Thompson J.B., Guggenheim S. (2002). Infrared Spectroscopy of Micas. Micas: Crystal Chemistry and Metamorphic Petrology.

[52] Zviagina B.B., Drits V.A., Dorzhieva O.V. (2020). Distinguishing Features and Identification Criteria for K-Dioctahedral 1M Micas (Illite-Aluminoceladonite and Illite-Glauconite-Celadonite Series) from Middle-Infrared Spectroscopy Data. Minerals.

[53] Meinhold R.H., MacKenzie K., Brown I.W.M. (1985). Thermal reactions of kaolinite studied by solid state 27-Al and 29-Si NMR. J. Mater. Sci. Lett..

[54] McKenzie K.J.D., Brown I.W.M., Meinhold R.H. (1985). Outstanding Problems in the Kaolinite-Mullite Reaction Sequence Investigated by 29-Si and 27-Al Solid-state Nuclear Magnetic Resonance: I, Metakaolonite. J. Am. Ceram. Chem..

[55] Sanz J., Serratosa J.M. (1984). 29-si and 27-al High Resolution MAS-NMR spectra of Phyllosolicates. J. Am. Chem. Soc..

[56] McKenzie K.J.D., Brown I.W.M., Cardile C.M., Meinhold R.H. (1987). The thermal reactions of muscovite studied by high-resolution solid-state 29-Si and 27-Al NMR. J. Mat. Sci..

[57] Smith J.V., Blackwell C.S., Hovis G.L. (1984). NMR of albite–microcline series. Nature.

[58] Monsif M., Zerouale A., Idrissi Kandri N., Mozzon M., Sgarbossa P., Zorzi F., Tateo F., Tamburini S., Franceschinis E., Carturan S. (2019). Chemical-physical and mineralogical characterization of ceramic raw materials from Moroccan regions: Intriguing resources for industrial applications. Appl. Clay Sci..

[59] Kothmann M.H., Ziadeh M., Bakis G., Rios de Anda A., Breu J., Altstadt V. (2015). Analyzing the influence of particle size and stiffness state of the annofiller on the mechanica properties of epoxy/clay nanocomposites using a novel shear-stiff nano-mica. J. Mater. Sci..

[60] Poh C.L., Mariatti M., Fauzi M.N.A., Ng C.H., Chee C.K., Chuah T.P. (2014). Tensile, dielectric, and thermal properties of epoxy composites filled with silica, mica, and calcium carbonate. J. Mater. Sci. Mater. Electron..

[61] Jouyandeh M., Akbari V., Paran S., Livi S., Lins L., Vahabi H., Saeb M. (2021). Epoxy/Ionic Liquid-Modified Mica Nanocomposites: Network Formation–Network Degradation Correlation. Nanomaterials.

[62] Loste J., Lopez-Cuesta J.M., Billon L., Garay H., Save M. (2019). Trasparent polymer nanocomposites: An overview on their synthesis and advanced properties. Prog. Polym. Sci..

[63] Trnka P., Mentlik V., Harvanek L., Hornak J., Matejka L. (2018). electrical and Thermo-mechanical Properties of DGEBA Cycloaliphatic Diamine Nano PA and SiO_2_ Composites. J. Elecr. Eng. Technol..

[64] Liu P., Xie Z., Pang X., Xu T., Zhang S., Morshuis P.H.F., Li H., Peng Z. (2022). Space Charge Behavior in Epoxy-Based Dielectrics: Progress and Perspective. Adv. Electron. Mater..

[65] Hashin Z., Shtrikman S. (1963). A variational approach to the theory of the elastic behaviour of multiphase materials. J. Mech. Phys. Solids.

[66] Quaresimin M., Bertani R., Zappalorto M., Pontefisso A., Simionato F., Bartolozzi A. (2015). Multifunctional polymer nanocomposites with enhanced mechanical and anti-microbial properties. Compos. Part B Eng..

[67] Zappalorto M., Salviato M., Pontefisso A., Quaresimin M. (2013). Notch effect in clay-modified epoxy: A new perspective on nanocomposite properties. Compos. Interfaces.

[68] Bertani R., Bartolozzi A., Pontefisso A., Quaresimin M., Zappalorto M. (2021). Improving the Antimicrobial and Mechanical Properties of Epoxy Resins via Nanomodification: An Overview. Molecules.

[69] Silling S., Askari E. (2005). A meshfree method based on the peridynamic model of solid mechanics. Comput. Struct..

[70] Seleson P. (2014). Improved one-point quadrature algorithms for two-dimensional peridynamic models based on analytical calculations. Comput. Methods Appl. Mech. Eng..

[71] Hill R. (1963). Elastic properties of reinforced solids: Some theoretical principles. J. Mech. Phys. Solids.

[72] Rafiee R., Shahzadi R. (2018). Mechanical Properties of Nanoclay and Nanoclay Reinforced Polymers: A Review. Polym. Compos..

[73] Wang K., Chen L., Wu J., Toh M.L., He C., Yee A.F. (2005). Epoxy Nanocomposites with Highly Exfoliated Clay: Mechanical Properties and Fracture Mechanisms. Macromolecules.

[74] Castellanos-Gomez A., Poot M., Amor-Amorós A., Steele G.A., Van Der Zant H.S.J., Agrait N., Rubio-Bollinger G. (2012). Mechanical properties of freely suspended atomically thin dielectric layers of mica. Nano Res..

[75] Gerstle W., Sau N., Silling S. (2007). Peridynamic modeling of concrete structures. Nucl. Eng. Des..

[76] Chen Z., Niazi S., Zhang G., Bobaru F., Voyiadjis G. (2018). Peridynamic Functionally Graded and Porous Materials: Modeling Fracture and Damage. Handbook of Nonlocal Continuum Mechanics for Materials and Structures.

[77] Ni T., Zaccariotto M., Zhu Q.-Z., Galvanetto U. (2019). Static solution of crack propagation problems in Peridynamics. Comput. Methods Appl. Mech. Eng..

