# Supplementary material for: Chemical and Mechanical Characterization of Unprecedented Transparent Epoxy–Nanomica Composites—New Model Insights for Mechanical Properties
Source: Polymers (Basel). 2023 Mar 15;15(6):1456. doi: 10.3390/polym15061456 (PMC10051337; doi:10.3390/polym15061456)

Supplementary Information

Chemical and mechanical characterization of unprecedented transparent epoxy-nanomica-composites. New model insights for mechanical properties

Greta Ongaro, Alessandro Pontefisso, Elena Zeni, Francesco Lanero, Alessia Famengo, Federico Zorzi, Mirco Zaccariotto, Ugo Galvanetto, Pietro Fiorentin, Renato Gobbo, Roberta Bertani, Paolo Sgarbossa

List of items:

- Table S1: Mineralogical composition of micas from XRD data
- Table S2: (a) Composition of Mica 10 by XRF data, (b) Composition of Mica 45 from XRF data
- Table S3: Values of the average transmittance for the tested nanocomposites
- Figure S1: FT IR spectra of Mica 10 and Mica 45 (a) in the 3000-4000 cm^{-1} region (b) and in 600-1250 cm^{-1} region
- Figure S2: ^{13}C Solid state NMR spectrum of Mica 45
- Figure S3: Particle size distribution of Mica 10 and Mica 45 powders
- Figure S4: ESEM images at different magnification of the neat epoxy resin and the EM composites
- Figure S5: T_{onset} decomposition as function of nominal filler weight percentages for Mica 10 and Mica 45 nanocomposites
- Figure S6: Residual mass (calculated from dry mass) at 700°C as function of nominal filler weight percentages for Mica 10 (void circles) and Mica 45 (full circles) nanocomposites

Table S1: Mineralogical composition of micas from XRD data

**Minerals (% wt)**	**Mica 10**	**Mica 45**
Quartz	13,1	9,9
Muscovite	62,7	45,1
Albite	4,1	3,4
Kaolinite	14,2	33,0
Microcline	5,9	8,6

Table S2:

(a) Composition of Mica 10 by XRF data

Element	Weight %	Atomic %
O	46,74	61,93
Na	0,48	0,44
Mg	0,72	0,69
Al	17,47	13,72
Si	24,57	18,55
K	7,63	4,13
Ti	0,6	0,26
Fe	2,52	0,95

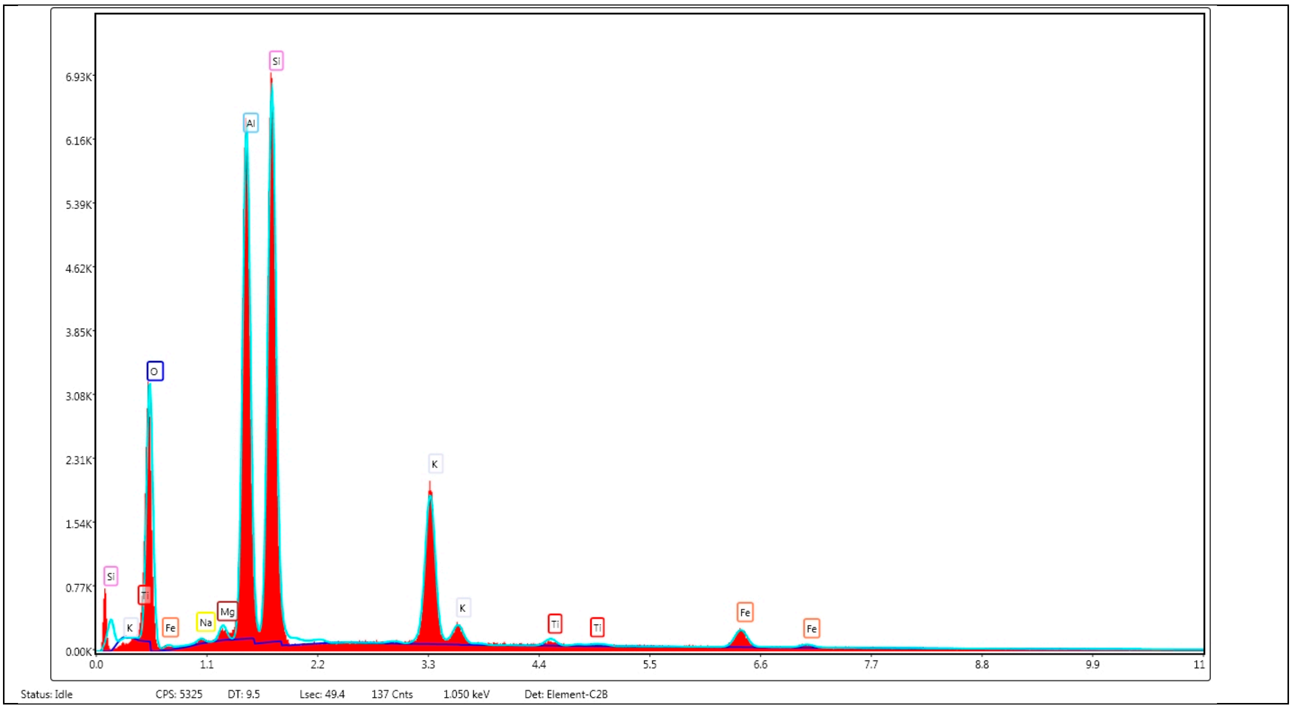

(b) Composition of Mica 45 from XRF data

Element	Weight %	Atomic %
Oxygen	47,31	62,09
Na	0,33	0,3
Mg	0,54	0,47
Al	18,7	14,55
Si	24,41	18,25
K	6,48	3,48
Ti	0,46	0,2
Fe	1,78	0,67

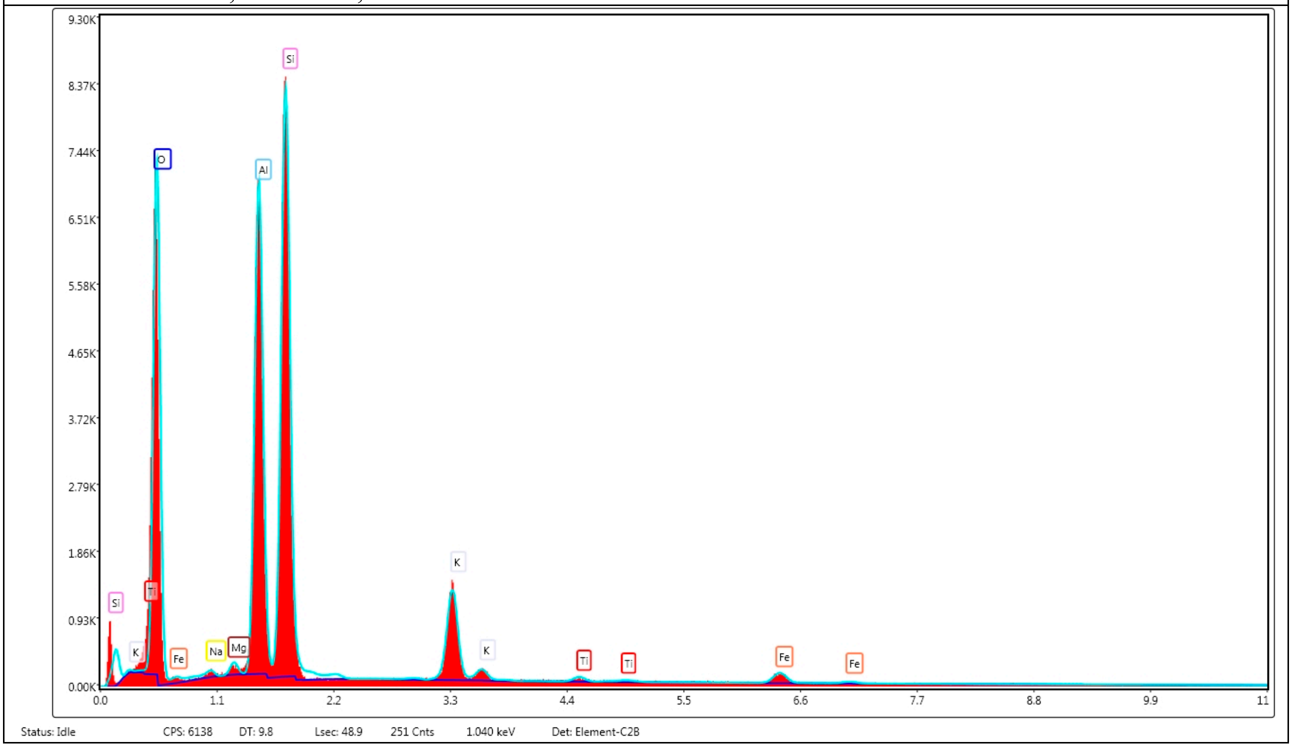

Table S3: Values of the average transmittance for the tested nanocomposites

	Mica amount (g) (%wt/wt)	Sample labeling	T (%)
**Neat**			**82**
**Mica 10**	1.6 g (1%)	EM10-1	**77**
	4.8 g (3%)	EM10-3	**75**
	8.0 g (5%)	EM10-5	**50**
**Mica 45**	1.6 g (1%)	EM45-1	**82**
	4.8 g (3%)	EM45-3	**73**
	8.0 g (5%)	EM45-5	**70**

The reported data are the average of two measurements collected under the same conditions.
The data can be compared with those reported in Huang, J; Nie, X. A simple and novel method to design flexible and transparent epoxy resin with tunable mechanical properties. *Polym. Int.* **2016**, *65*, 835-840.

Figure S1: FT IR spectra of Mica 10 and Mica 45

(a) in the 3000-4000 cm^{-1} region

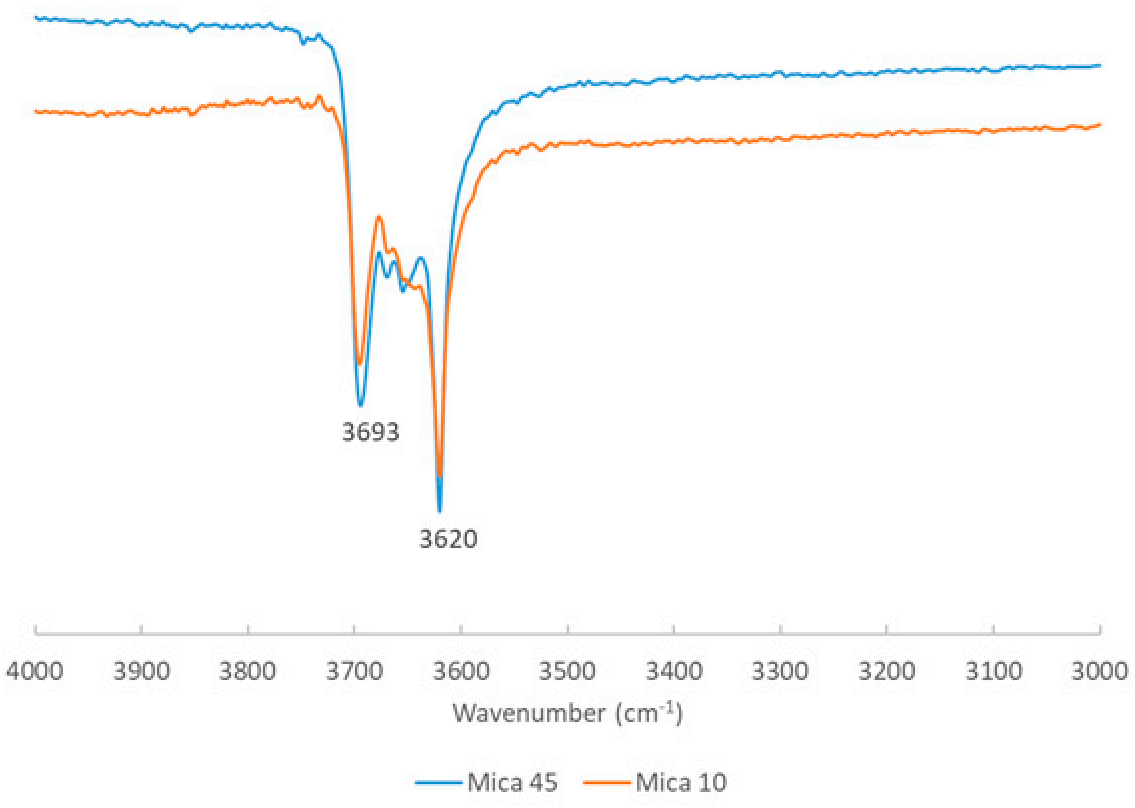

(b) and in 600-1250 cm^{-1} region

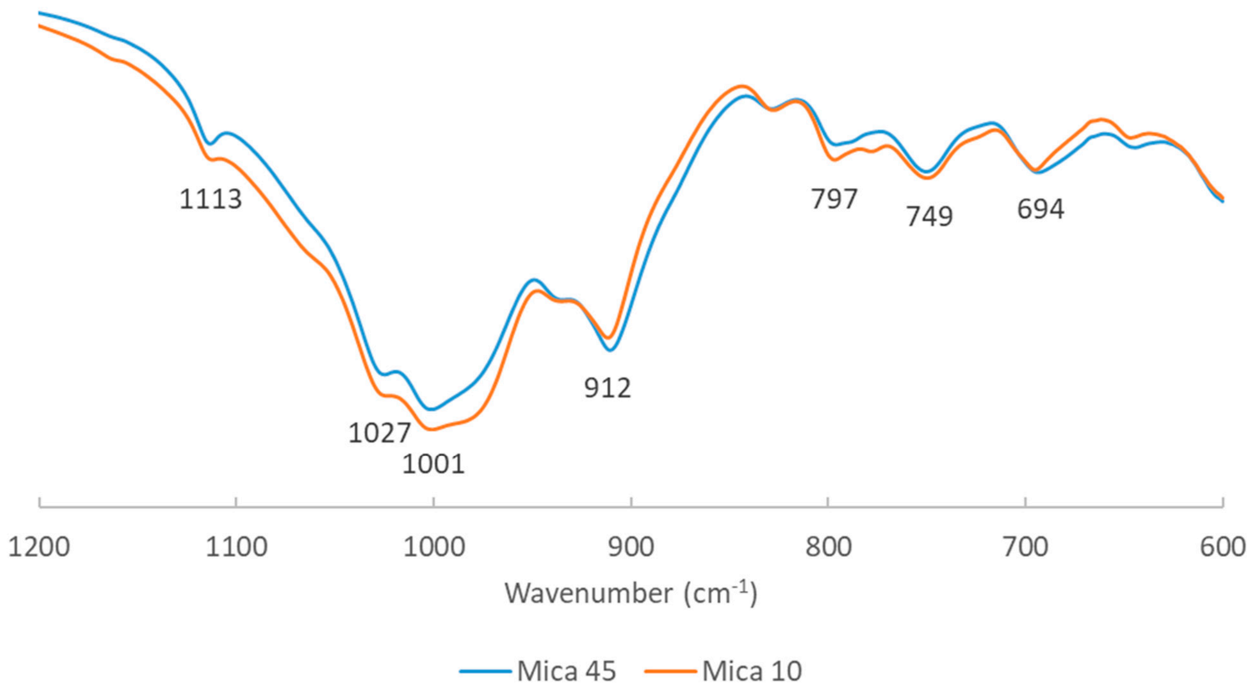

Figure S2: ^{13}C Solid state NMR spectrum of Mica 45

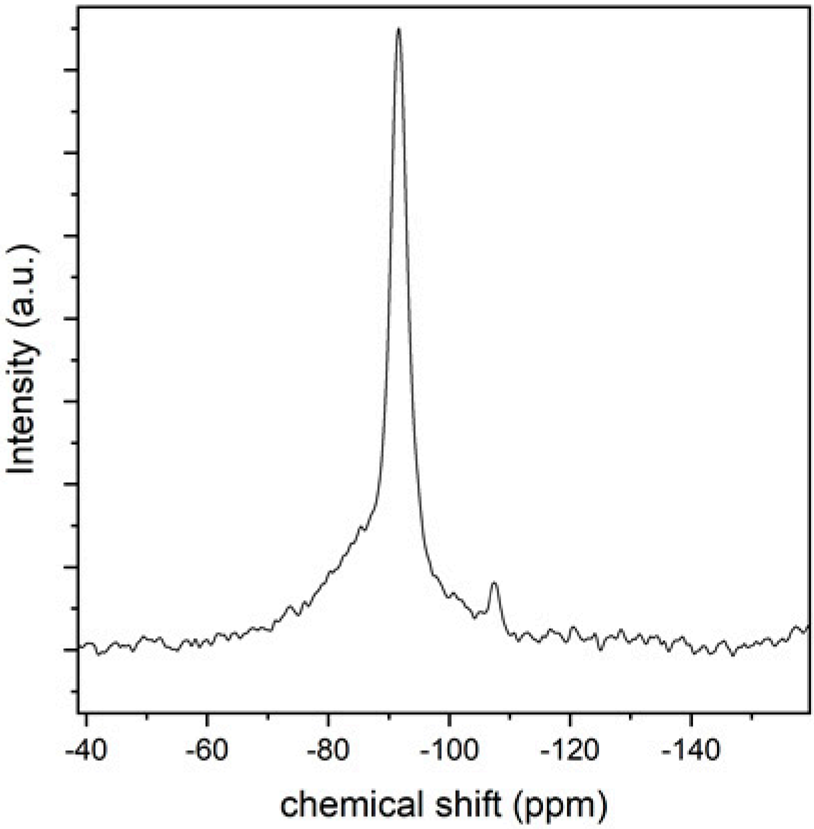

Figure S3: Particle size distribution of Mica 10 and Mica 45 powders

MASTERSIZER 2000

Result Analysis Report

**Sample Name:**<br>MICA 10 - Average	**SOP Name:**	**Measured:**<br>venerdì 14 ottobre 2022 08:46:39
**Sample Source & type:**	**Measured by:**<br>laboratorio	**Analysed:**<br>venerdì 14 ottobre 2022 08:46:40
**Sample bulk lot ref:**	**Result Source:**<br>Averaged	

**Particle Name:**<br>Silica 0.1	**Accessory Name:**<br>Hydro 2000SM (A)	**Analysis model:**<br>Multiple narrow modes	**Sensitivity:**<br>Enhanced
**Particle RI:**<br>1.544	**Absorption:**<br>0.1	**Size range:**<br>0.020 to 2000.0... um	**Obscuration:**<br>14.50 %
**Dispersant Name:**<br>Ethanol	**Dispersant RI:**<br>1.360	**Weighted Residual:**<br>0.996 %	**Result Emulation:**<br>Off

**Concentration:**<br>0.0117 %Vol	**Span :**<br>1.604	**Uniformity:**<br>0.506	**Result units:**<br>Volume
**Specific Surface Area:**<br>1.2 m²/g	**Surface Weighted Mean D[3,2]:**<br>4.984 um	**Vol. Weighted Mean D[4,3]:**<br>9.304 um	

d(0.1): 2.823 um   d(0.5): 8.539 um   d(0.9): 16.521 um

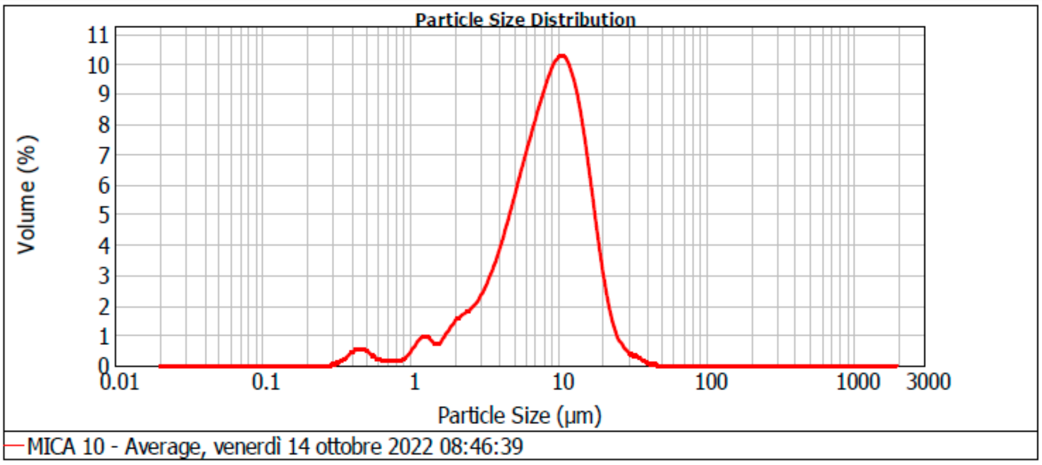

Size (µm)	Volume In %	Size (µm)	Volume In %	Size (µm)	Volume In %	Size (µm)	Volume In %	Size (µm)	Volume In %	Size (µm)	Volume In %
0.010	0.00	0.105	0.00	1.096	0.79	11.482	8.83	120.226	0.00	1258.925	0.00
0.011	0.00	0.120	0.00	1.259	0.80	13.183	7.67	138.038	0.00	1445.440	0.00
0.013	0.00	0.138	0.00	1.445	0.63	15.136	5.94	158.489	0.00	1659.587	0.00
0.015	0.00	0.158	0.00	1.660	0.96	17.378	3.92	181.970	0.00	1905.461	0.00
0.017	0.00	0.182	0.00	1.905	1.33	19.953	2.18	208.930	0.00	2187.762	0.00
0.020	0.00	0.209	0.00	2.188	1.56	22.909	1.03	239.883	0.00	2511.886	0.00
0.023	0.00	0.240	0.00	2.512	1.80	26.303	0.47	275.423	0.00	2884.032	0.00
0.026	0.00	0.275	0.02	2.884	2.22	30.200	0.28	316.228	0.00	3311.311	0.00
0.030	0.00	0.316	0.14	3.311	2.83	34.674	0.14	363.078	0.00	3801.894	0.00
0.035	0.00	0.363	0.33	3.802	3.60	39.811	0.02	416.869	0.00	4365.158	0.00
0.040	0.00	0.417	0.49	4.365	4.53	45.709	0.00	478.630	0.00	5011.872	0.00
0.046	0.00	0.479	0.41	5.012	5.51	52.481	0.00	549.541	0.00	5754.399	0.00
0.052	0.00	0.550	0.20	5.754	6.50	60.256	0.00	630.957	0.00	6606.934	0.00
0.060	0.00	0.631	0.13	6.607	7.43	69.183	0.00	724.436	0.00	7585.776	0.00
0.069	0.00	0.724	0.11	7.586	8.31	79.433	0.00	831.764	0.00	8709.636	0.00
0.079	0.00	0.832	0.17	8.710	9.00	91.201	0.00	954.993	0.00	10000.000	
0.091	0.00	0.955	0.44	10.000	9.26	104.713	0.00	1096.478	0.00		
0.105		1.096		11.482		120.226		1258.925			

MALVERN INSTRUMENTS

MASTERSIZER 2000

Result Analysis Report

Sample Name:
MICA 145 - Average

SOP Name:

Measured:
venerdì 14 ottobre 2022 08:58:38

Sample Source & type:

Measured by:
laboratorio

Analysed:
venerdì 14 ottobre 2022 08:58:39

Sample bulk lot ref:

Result Source:
Averaged

Particle Name:
Silica 0.1

Accessory Name:
Hydro 2000SM (A)

Analysis model:
Multiple narrow modes

Sensitivity:
Enhanced

Particle RI:
1.544

Absorption:
0.1

Size range:
0.020 to 2000.0... um

Obscuration:
15.69 %

Dispersant Name:
Ethanol

Dispersant RI:
1.360

Weighted Residual:
0.630 %

Result Emulation:
Off

Concentration:
0.0268 %Vol

Span :
1.938

Uniformity:
0.621

Result units:
Volume

Specific Surface Area:
0.51 m²/g

Surface Weighted Mean D[3,2]:
11.776 um

Vol. Weighted Mean D[4,3]:
22.185 um

d(0.1): 5.963 um

d(0.5): 18.616 um

d(0.9): 42.049 um

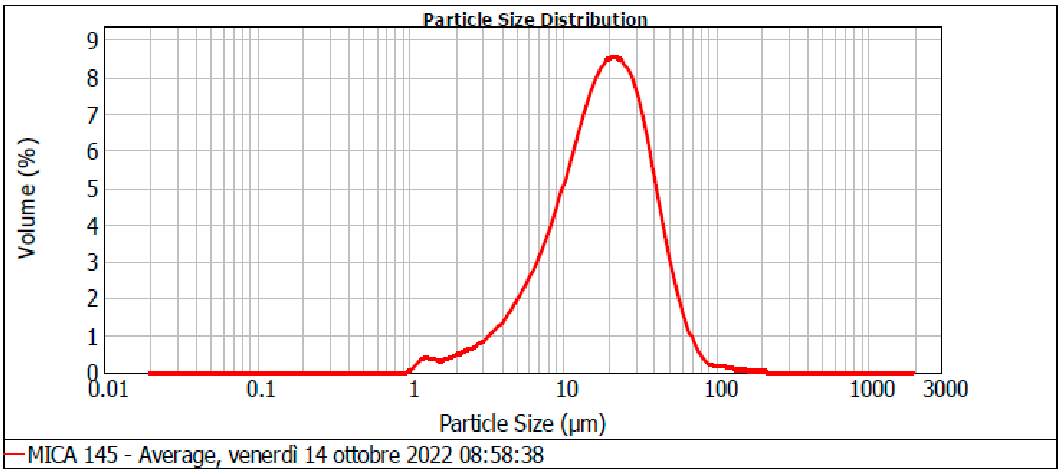

Size (µm)	Volume In %
0.010	0.00
0.011	0.00
0.013	0.00
0.015	0.00
0.017	0.00
0.020	0.00
0.023	0.00
0.026	0.00
0.030	0.00
0.035	0.00
0.040	0.00
0.046	0.00
0.052	0.00
0.060	0.00
0.069	0.00
0.079	0.00
0.091	0.00
0.105	

Size (µm)	Volume In %
0.105	0.00
0.120	0.00
0.138	0.00
0.158	0.00
0.182	0.00
0.209	0.00
0.240	0.00
0.275	0.00
0.316	0.00
0.363	0.00
0.417	0.00
0.479	0.00
0.550	0.00
0.631	0.00
0.724	0.00
0.832	0.00
0.955	0.05
1.096	

Size (µm)	Volume In %
1.096	0.30
1.259	0.34
1.445	0.29
1.660	0.34
1.905	0.43
2.188	0.53
2.512	0.63
2.884	0.77
3.311	0.98
3.802	1.25
4.365	1.58
5.012	1.95
5.754	2.36
6.607	2.83
7.586	3.40
8.710	4.09
10.000	4.89
11.482	

Size (µm)	Volume In %
11.482	5.70
13.183	6.48
15.136	7.11
17.378	7.53
19.953	7.71
22.909	7.62
26.303	7.22
30.200	6.46
34.674	5.39
39.811	4.16
45.709	2.99
52.481	2.01
60.256	1.22
69.183	0.63
79.433	0.28
91.201	0.16
104.713	0.14
120.226	

Size (µm)	Volume In %
120.226	0.10
138.038	0.06
158.489	0.04
181.970	0.03
208.930	0.00
239.883	0.00
275.423	0.00
316.228	0.00
363.078	0.00
416.869	0.00
478.630	0.00
549.541	0.00
630.957	0.00
724.436	0.00
831.764	0.00
954.993	0.00
1096.478	0.00
1258.925	

Size (µm)	Volume In %
1258.925	0.00
1445.440	0.00
1659.587	0.00
1905.461	0.00
2187.762	0.00
2511.886	0.00
2884.032	0.00
3311.311	0.00
3801.894	0.00
4365.158	0.00
5011.872	0.00
5754.399	0.00
6606.934	0.00
7585.776	0.00
8709.636	0.00
10000.000	

Figure S4: ESEM images at different magnification of the neat epoxy resin and the EM composites

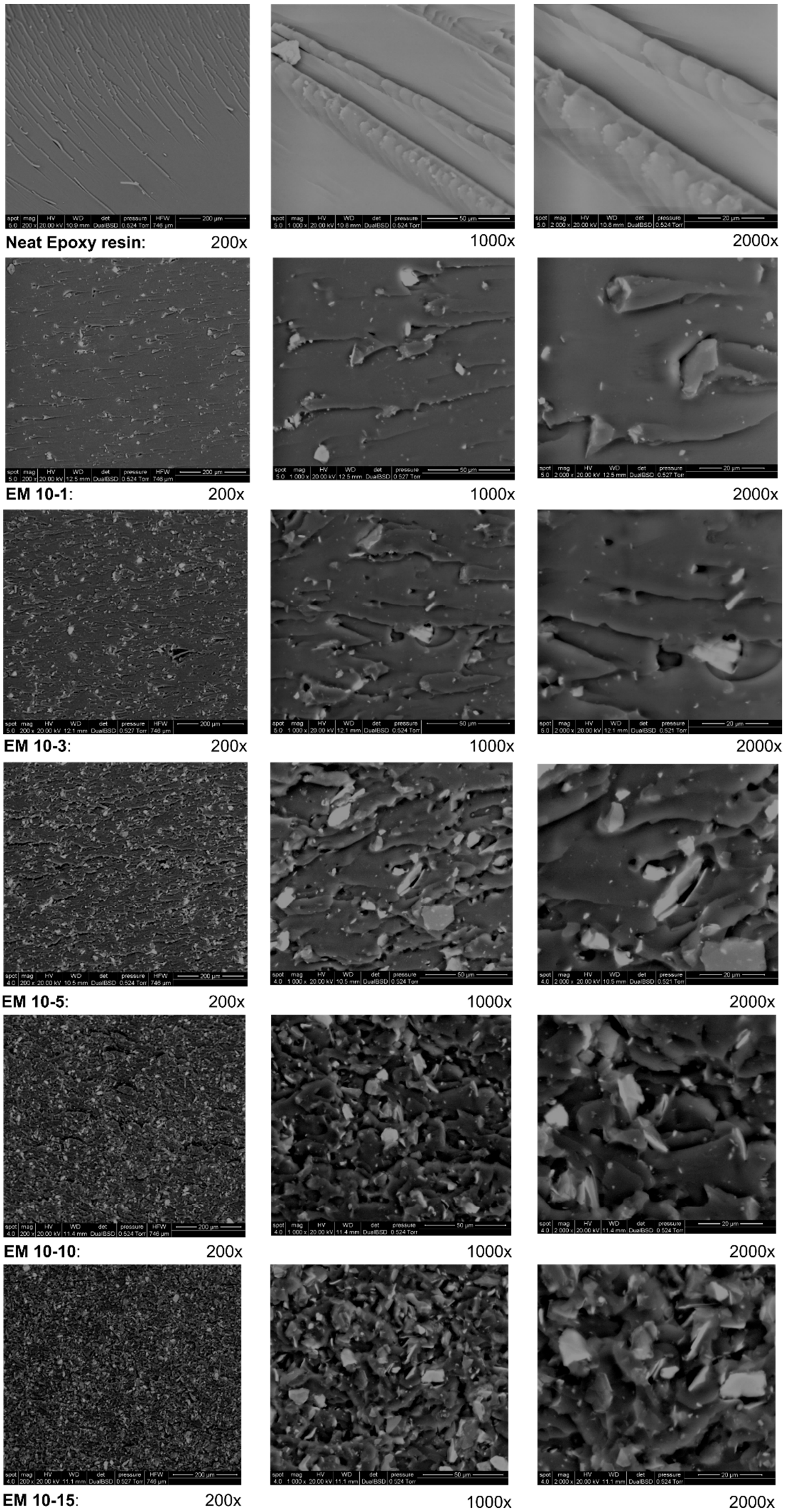

Figure S5: T_{onset} decomposition as function of nominal filler weight percentages for Mica 10 and Mica 45 nanocomposites

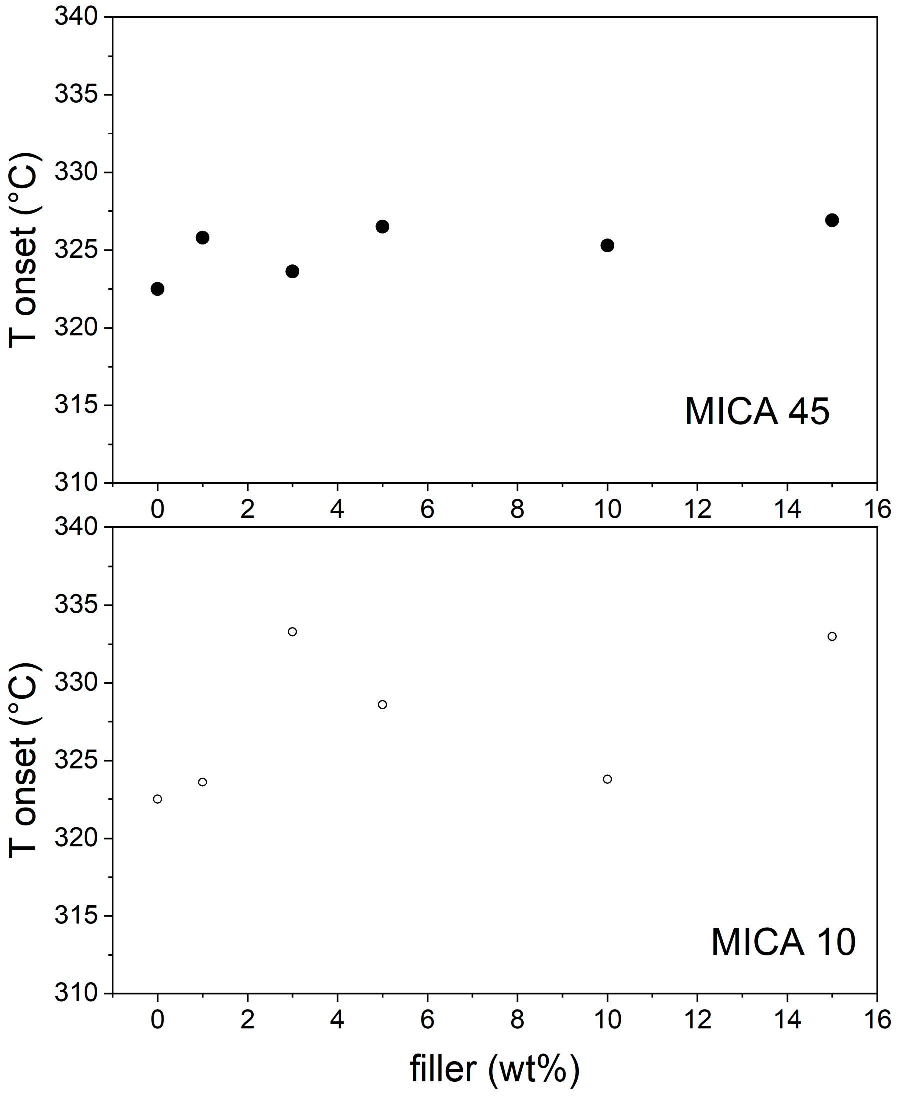

Figure S6: Residual mass (calculated from dry mass) at 700°C as function of nominal filler weight percentages for Mica 10 (void circles) and Mica 45 (full circles) nanocomposites

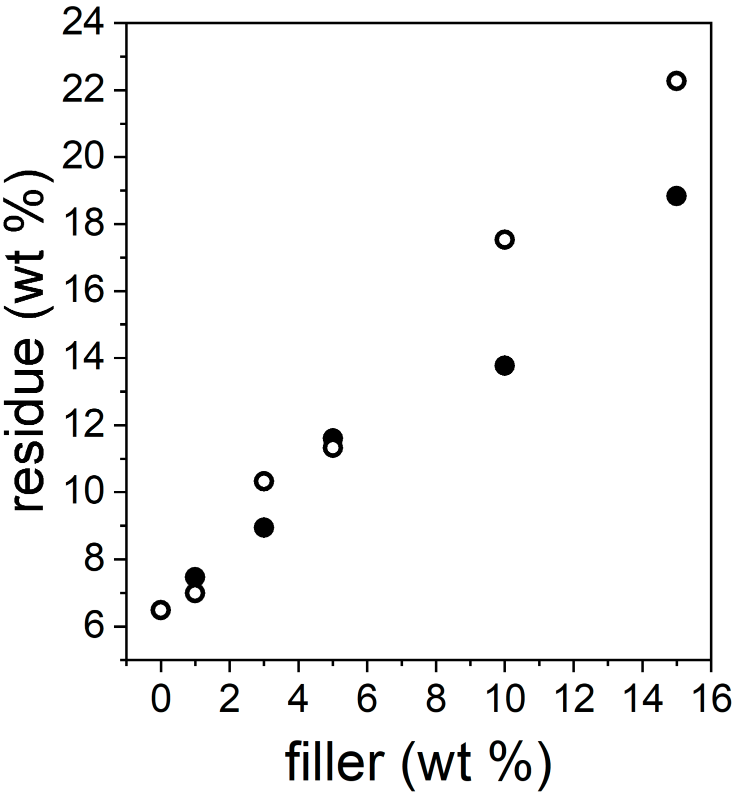

Supplement: Supplementary file 1 [file polymers-15-01456-s001.zip › polymers-2253666-supplementary.pdf]
